# Performance of Recycled Concrete Aggregate and Reclaimed Asphalt Pavement in Concrete: A Systematic Review of Mechanical, Physical, and Durability Characteristics

**DOI:** 10.3390/ma19173601

**Published:** 2026-08-25

**Authors:** Ahmed Ashteyat, Aye Alkhalaileh, Mousa Shhabat, Hebah Al-zu’bi, Sultan Almuaythir, Mahmoud Nawasreh

**Affiliations:** 1 Department of Civil Engineering, College of Engineering in Al-Kharj, Prince Sattam Bin Abdulaziz University, Al-Kharj 11942, Saudi Arabia; a.ashteyat@psau.edu.sa (A.A.); s.alhomair@psau.edu.sa (S.A.); 2 Department of Civil Engineering, Al-Balqa Applied University, Al-Salt 19117, Jordan; alkhalailehaye@gmail.com; 3 Department of Civil Engineering, Faculty of Engineering, The University of Jordan, Amman 11942, Jordan; 4 Civil and Environmental Engineering, King Fahd University of Petroleum and Minerals, Dhahran 31261, Saudi Arabia; hbh8190831@ju.edu.sa; 5 Department of Mathematics, College of Science and Humanities, Prince Sattam Bin Abdulaziz University, Al-Kharj 11942, Saudi Arabia; m.nawasreh@psau.edu.sa

**Keywords:** recycled concrete aggregate (RCA), recycled asphalt pavement (RAP), mechanical behavior, physical behavior, concrete durability

## Abstract

The increasing generation of construction and demolition waste, along with the depletion of natural aggregates, has driven growing interest in recycled concrete aggregate (RCA) and reclaimed asphalt pavement (RAP) as sustainable alternatives in concrete production. However, a direct and systematic comparison between the two materials remains limited. This review addresses this gap by applying PRISMA guidelines to analyze 82 peer-reviewed studies published between 2010 and 2026. Both materials are evaluated across three key domains: physical properties, mechanical performance, and microstructural characteristics. The findings indicate that RCA can reduce compressive strength by up to 26%, mainly due to the presence of porous adhered mortar and a complex interfacial transition zone (ITZ). In contrast, RAP weakens bonding with cement paste because of its hydrophobic bituminous coating, leading to adhesive failure at the mortar asphalt interface. Despite these limitations, RCA and RAP exhibit distinct behaviors in terms of shear capacity, ductility, energy absorption, and durability. Enhancement techniques such as surface treatment, carbonation, supplementary cementitious materials, and fiber reinforcement show potential in improving performance. Additionally, life cycle and economic analyses reveal that RAP can reduce total costs and carbon emissions when efficiently processed. This study provides a unified comparative framework to support sustainable material selection and design optimization.

## 1. Introduction

The demolition of end-of-life structures and the management of construction and demolition waste (C&DW) represent two of the most pressing environmental challenges in modern infrastructure development. The USA alone generates approximately 317 million tons of C&DW annually, while European countries collectively produce around 510 million tons per year [1,2]. The construction industry as a whole produces roughly 900 million tons of solid waste each year, primarily concrete, whose recycling has been identified as necessary to meet legislative targets such as the European 70% inert waste recycling objective and to reduce the burden on increasingly constrained landfills [3]. Alongside this waste crisis, the rapid depletion of natural aggregate reserves intensifies the urgency for recycled alternatives. Aggregate cost comprises 20–30% of total material cost in concrete pavement, and many high-quality aggregate sources have already been exhausted, driving continuous increases in purchasing costs [4]. The scarcity of natural aggregates and the absence of eco-friendly construction materials have accelerated global interest in utilizing recycled aggregates as a partial or full substitute for virgin materials in structural and pavement applications [5]. The production of concrete components also generates enormous quantities of CO_2_, making the recycling of construction debris a requisite measure for achieving sustainable progress in the construction sector [6]. Among the materials recoverable from C&DW streams, recycled concrete aggregate (RCA) and reclaimed asphalt pavement (RAP) stand out as the two most abundant and structurally consequential by-products. Both have been identified as suitable sources for producing recycled aggregate concrete, attracting considerable research interest as alternatives to natural coarse aggregate [2]. Recycled aggregates can be classified by particle size into recycled concrete aggregate (RCA, >4.75 mm), recycled fine aggregate (RFA, 0.16–4.75 mm), and recycled powder (RP, <0.16 mm), with RCA constituting approximately 50% of total C&DW [7]. Although RAP is traditionally recycled into new asphalt mixtures and remains the preferred reuse pathway for pavement engineering, its utilization in cementitious materials has attracted increasing research interest because large quantities of reclaimed asphalt continue to be generated worldwide, while not all RAP can be economically or technically reincorporated into asphalt production. Consequently, alternative high-value applications have been explored to increase recycling rates and reduce dependence on natural aggregates. Unlike RCA, RAP contains a residual bituminous coating that fundamentally alters its interaction with cement paste, resulting in different interfacial mechanisms, failure modes, and mechanical behavior. Therefore, comparing RCA and RAP is not intended to demonstrate their equivalence but rather to establish a mechanistic understanding of how two abundant recycled aggregate streams perform differently in concrete and to identify the applications and enhancement strategies most suitable for each material.

Despite their shared origin in C&DW, the engineering behaviors of RCA and RAP differ in fundamental ways that demand systematic comparative investigation. RCA inherently exhibits inferior mechanical performance relative to natural aggregate, primarily due to the residual mortar layer adhered to its surface. This layer introduces elevated porosity, higher water absorption, surface microcracking, and a reduced modulus of elasticity, all of which negatively affect structural integrity and durability [8]. Full RCA replacement can decrease compressive strength by up to 26%, with smaller losses achieved at lower water-to-binder ratios or when higher-strength parent concrete is used [9]. Surface treatment strategies such as water glass immersion and mechanical strengthening have demonstrated effectiveness in upgrading interfacial transition zone (ITZ) quality and recovering mechanical performance [10,11]. RAP aggregates present a fundamentally different challenge. The aged bituminous binder coating their mineral surface is hydrophobic, limiting cement paste adhesion and producing a thicker, more porous (ITZ) than that observed in natural aggregate concrete [12]. Failure mechanisms in RAP concrete are governed by aggregate mineralogy, mortar grade, and asphalt condition shifting between cohesive and adhesive modes in ways that have no direct parallel in RCA systems [12]. At the microstructural level, the ITZ between aggregate and paste is the critical zone controlling both strength and durability in concrete incorporating either material; however, its chemistry, morphology, and modifiability differ markedly between RCA and RAP, with direct consequences for mechanical response and surface treatment effectiveness [8,10]. In structural applications, RCA and RAP produce meaningfully different responses in shear and flexural behavior, crack patterns, reserved strength, and energy absorption including under elevated temperature conditions [2,13,14].

In pavement applications, the two materials similarly exhibit opposing effects on mix performance: RAP can enhance resilient modulus at higher stress levels [15], while RCA alters air voids and optimum asphalt demand in hot-mix systems [16]. From a sustainability perspective, life cycle assessments confirm that RAP offers favorable economic benefits relative to both natural aggregate and RCA in targeted pavement applications [4,17], while mineral admixtures and optimized mix design can compensate for mechanical losses in both recycled aggregate systems [5,18,19]. Despite the extensive individual literature on RCA and RAP, a systematic side-by-side comparison of their mechanical, physical, and microstructural characteristics within a unified analytical framework remains limited. Existing reviews address RAP in pavement construction [5,20] or RCA in structural concrete in isolation [3], while structural studies involving both materials simultaneously [2,14] confirm that their responses in crack patterns, reserved strength, and energy absorption are distinct and require direct comparative investigation. The present review addresses this gap by consolidating findings for both materials across three domains, (i) physical properties, (ii) mechanical performance, and (iii) microstructural characteristics, with the aim of identifying knowledge gaps and guiding future research into the optimized co-utilization of both recycled materials in sustainable construction.

## 2. Research Significance

Although RCA and RAP both originate from construction and demolition waste, the existing literature addresses each material in isolation, focusing either on RCA in structural concrete or RAP in pavement engineering, without reconciling their behaviors within a unified comparative framework. No study systematically contrasts the mechanical, physical, and microstructural characteristics of both materials across identical evaluation domains, leaving engineers without a reliable basis for selecting between them or assessing combined RCA–RAP applications. This gap is consequential as global demolition activity generates hundreds of millions of tons of waste annually, creating surpluses of both materials that demand clear, evidence-based utilization pathways. Furthermore, RCA and RAP differ fundamentally in interfacial behavior: RCA introduces a double ITZ from adhered cement mortar, and RAP presents a hydrophobic asphalt-coated surface, meaning performance penalties and optimal applications are material-specific and cannot be generalized from one to the other. To the authors’ knowledge, this review is the first to directly compare RCA and RAP across three defined domains, physical properties, mechanical performance, and microstructural characteristics, evaluated using the same criteria applied equally to both materials. It establishes a unified framework correlating macro-scale behavior with microstructural observations, enabling a mechanistic understanding of their differing performance in cementitious and pavement systems. It further synthesizes enhancement strategies including carbonation modification, surface treatment, and fiber reinforcement mapped specifically to each material. This consolidated reference supports informed material selection and optimized mix design for both recycled aggregate systems. It further provides a foundation for developing design standards that account for the distinct mechanical and microstructural characteristics of RCA and RAP, benefiting engineers, researchers, and policymakers engaged in sustainable construction. Importantly, this comparison does not advocate for replacing the conventional use of RAP in asphalt pavements, where it remains the preferred and most established recycling pathway. Instead, it evaluates RAP as an alternative utilization route in cementitious materials in situations where asphalt recycling is impractical or surplus RAP is available. The objective is to identify the distinct engineering limitations, failure mechanisms, and enhancement strategies associated with RAP and RCA, thereby providing engineers with a rational basis for selecting the most appropriate recycled aggregate for specific concrete applications.

## 3. Methodology

The methodological framework adopted in this comparative review follows the Preferred Reporting Items for Systematic Reviews and Meta-Analyses (PRISMA) guidelines to ensure transparency, reproducibility, and methodological rigor in the identification, screening, and synthesis of relevant studies. This systematic review was conducted and reported in accordance with the Supplementary Materials PRISMA 2020 Statement [21]. This systematic review was not prospectively registered; therefore, no registration number is available. Furthermore, no formal review protocol was prepared prior to conducting this review.

The objective of this systematic review was to critically evaluate and synthesize research published between 2010 and 2026 concerning the mechanical, physical, durability, and microstructural characteristics of recycled concrete aggregate (RCA) and reclaimed asphalt pavement (RAP) in construction materials. The literature search was performed using six major scientific databases, namely Scopus, Web of Science, ScienceDirect, Springer, Wiley-Blackwell, and MDPI. A structured search strategy based on Boolean operators was adopted to maximize both the relevance and comprehensiveness of the retrieved studies. The search terms were organized into five principal thematic groups covering the following: (i) material type (RCA and RAP); (ii) engineering performance (mechanical, physical, and durability properties); (iii) microstructural characteristics; (iv) enhancement strategies; and (v) construction applications.

The eligibility criteria required that studies be peer-reviewed journal articles published in English between 2010 and 2026, directly addressing the performance of RCA and/or RAP in construction materials. Both experimental investigations and high-quality review articles were considered when they provided substantial technical insights. Studies were excluded if they were not directly related to RCA or RAP, focused solely on economic or life cycle assessment without evaluating engineering performance, investigated unrelated recycled materials, or consisted of conference abstracts or reports lacking sufficient experimental or analytical information. To ensure meaningful comparisons between RCA and RAP, priority was given to studies conducted under comparable experimental conditions, including similar concrete strength grades, water-to-binder ratios, curing regimes, and testing standards. Whenever available, studies directly comparing RCA and RAP within the same experimental program were considered the primary source of evidence.

The study selection procedure followed the four stages recommended by the PRISMA 2020 framework, namely identification, screening, eligibility, and inclusion. During the eligibility stage, studies were evaluated using predefined quality assessment criteria according to their study type. Experimental investigations were assessed based on the clarity of specimen descriptions, testing procedures, and consistency of reported mechanical and microstructural findings. Review articles were evaluated according to the comprehensiveness of the literature coverage, quality of synthesis, and relevance to the objectives of this review, whereas pavement and structural studies were assessed with respect to the transparency of material characterization, mixture design, and performance evaluation methods.

Data extraction was performed using a standardized review framework to ensure consistency across all included studies. For each eligible article, information regarding publication year, recycled material type, replacement level, material properties, experimental methodology, testing conditions, and the reported mechanical, durability, and microstructural performance was systematically recorded. The extracted data were subsequently synthesized through a qualitative comparative analysis to identify common trends, inconsistencies, knowledge gaps, and research opportunities related to the comparative performance of RCA- and RAP-based construction materials. Owing to the substantial heterogeneity among the included studies with respect to aggregate source, mixture proportions, curing conditions, specimen geometry, and testing procedures, a quantitative meta-analysis was not considered appropriate. Consequently, the findings were synthesized using a qualitative comparative approach.

## 4. Study Selection and Characteristics of the Included Studies

**Figure 1:**
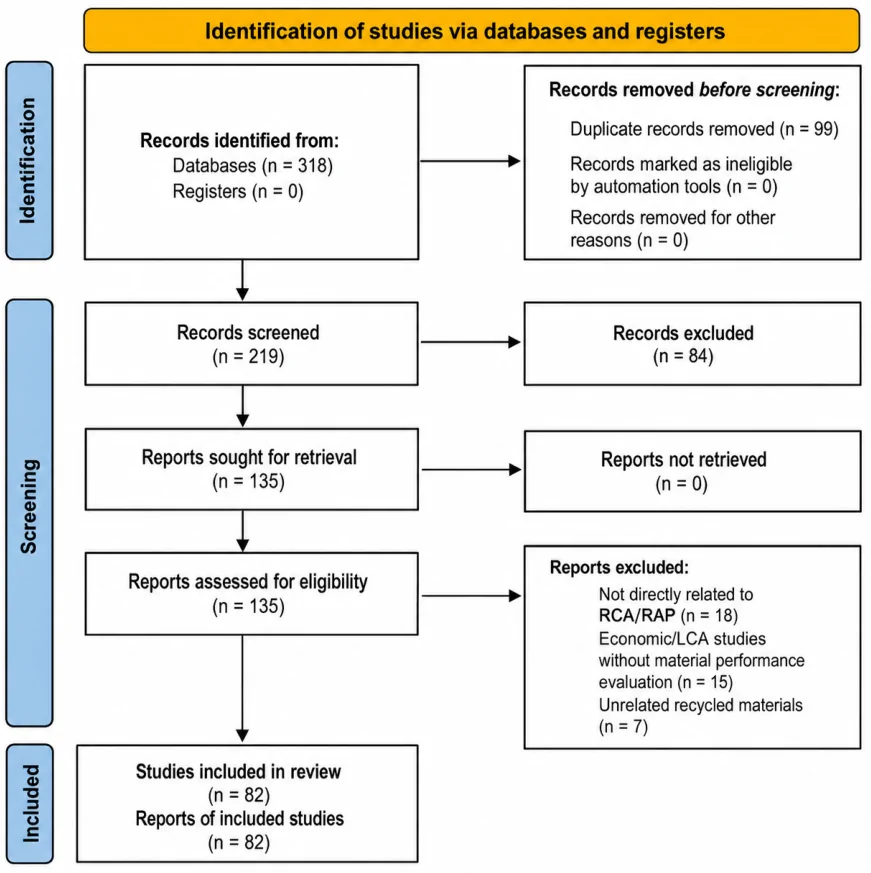
PRISMA 2020 flow diagram illustrating the study selection process.

A comprehensive literature search was conducted across six scientific databases, including Scopus, Web of Science, ScienceDirect, Springer, Wiley-Blackwell, and MDPI, resulting in the identification of 318 records. After removing 99 duplicate records, 219 unique studies remained and were screened based on their titles and abstracts. Subsequently, 135 full-text articles were retrieved and assessed for eligibility. All selected articles were successfully obtained for full-text evaluation; therefore, no reports were excluded because of unavailable full texts. Following the eligibility assessment, 53 full-text articles were excluded for predefined methodological and relevance-related reasons, as summarized in Figure 1. Ultimately, 82 studies satisfied all inclusion criteria and were included in the final qualitative synthesis. The complete study selection process is illustrated in Figure 1. Detailed information for all included studies is provided in Appendix A (Table A1).

## 5. Properties of Recycled Concrete Aggregate (RCA) and Reclaimed Asphalt Pavement (RAP)

Table 1 presents a comparative summary of the typical physical and mechanical properties of RCA and RAP as reported across the reviewed literature. The properties of RCA show higher variability due to differences in source concrete quality, demolition method, and degree of processing. RAP properties are influenced by the type and degree of aging of the asphalt binder, aggregate mineralogy, and the percentage of binder content retained in the reclaimed material. RCA typically exhibits lower specific gravity than natural aggregates due to the presence of porous adhered mortar, with SSD values generally ranging from 2.16 to 2.53 compared to 2.55–2.70 for NA, a reduction of 2–15%. Water absorption is substantially higher, typically ranging from 1.50% to 9.00% versus 0.28–2.00% for NA, which reflects the porosity of the mortar coating and has direct implications for effective water-to-cement-ratio control in mix design. The Los Angeles abrasion loss of RCA ranges from 28% to 45%, substantially exceeding the 15–30% typical of NA, indicating reduced abrasion resistance that varies with source concrete strength and degree of mortar removal. The interfacial transition zone in RCA-based concrete is particularly complex, forming a so-called “double ITZ” at both the old mortar–new cement pastes interface and the mortar original aggregate interface, with ITZ thickness reaching 20–50 µm compared to 5–20 µm in natural aggregate concrete, creating two planes of weakness that govern crack initiation and propagation. RAP aggregates exhibit moderate specific gravity, with SSD values ranging from 2.20 to 2.49, and relatively lower water absorption of 0.20–3.50% compared to RCA, though total open porosity can be elevated (2.0–8.0%) depending on the degree of asphalt aging and stripping. A defining characteristic of RAP is the residual asphalt binder content, which typically ranges from 2.17% to 5.60% by mass depending on particle fraction. This binder coating reduces the wettability and adhesion capacity of the aggregate surface in cementitious applications, RAP surfaces exhibit contact angles significantly exceeding 90°, confirming hydrophobic behavior [12,22], thereby weakening the ITZ and shifting the dominant failure mode toward adhesion at the mortar–asphalt interface. In asphalt applications, however, the aged binder can contribute to mixture stiffness and stability when properly blended with virgin binder [16]. The particle shape of RAP tends to be angular to sub-angular with a flakiness index below 10% (approximately 6% elongated particles per ASTM D4791) [15,23,24,25] though irregular fragments resulting from milling operations can affect workability and compaction in both concrete and pavement mixtures [8]. As summarized in Table 1, the physical property differences between RCA and RAP are visually apparent. Table 1 shows that water absorption increases markedly with RCA replacement ratio from 1.50% up to 9.00% at full replacement, far exceeding that of RAP (0.20–3.50%) due to the porous adhered mortar, while RAP maintains relatively lower absorption owing to its hydrophobic asphalt coating. Table 1 compares the specific gravity ranges of both materials against natural aggregate, confirming that RCA (2.16–2.53) is consistently lighter than both NA (2.55–2.70) and RAP (2.20–2.49), a direct consequence of the lower-density mortar layer, while RAP falls closer to natural aggregate values due to the denser mineral aggregate beneath the binder coating. 

## 6. Effect of RCA and RAP on Concrete’s Mechanical and Structural Behavior

### 6.1. Compressive Strength 

The compressive strength (CS) of concrete is widely regarded as the primary indicator of structural performance, and it is consistently affected to varying degrees by the incorporation of RCA and RAP. For RCA used in ultra-high-performance concrete (UHPC), the nature and extent of CS change depend critically on the treatment applied to the aggregate surface. Incorporating untreated recycled fine aggregate (RFA) progressively reduced CS with increasing replacement ratios. However, carbonation treatment of RCA/RFA has demonstrated a notable reversal of this trend. Carbonated recycled coarse aggregate (CRCA) incorporated into UHPC achieved a 28-day CS of 122.66 MPa, representing a 9.1% gain over the natural aggregate baseline [47]; similarly, 20% carbonated RFA produced 28-day CS values equivalent to plain UHPC, whereas the same proportion of uncarbonated RFA reduced CS by 12% [7]. At the microwave-assisted carbonation level, an optimal 50% replacement of silica fume with microwave-carbonated recycled concrete fines (MCRCFs) increased the 28-day CS of UHPC by 3.9% [62]. Replacement of cement with recycled concrete powder (RCP) showed a non-linear trend: 10% and 20% RCP increased CS slightly (by 4.4% and 1.2%, respectively), while 70% RCP caused a 17.6% reduction, from a baseline of 157.6 MPa down to 129.9 MPa [63]. Particle size selection was also shown to govern CS outcomes; using RFA of the 1.18–2.36 mm fraction at 20% replacement increased 28-day CS by 7.1%, reaching 160.6 MPa [58]. When RCA was introduced alongside carbon nanofibers (CNFs) and steel fibers (SFs) in UHPC, even 50% RCA replacement caused only a slight 3% CS reduction at 28 days, with hybrid CNF–SF addition recovering and even surpassing the control, reaching 180.9 MPa [64]. The addition of graphene oxide (GO) at 0.06 wt% to UHPC with 100% fine RA further raised CS by up to 16.04%, bringing it above the natural-sand reference mix [65]. In contrast, RAP aggregate, owing to the hydrophobic asphalt film coating its particles, consistently and more severely impairs CS regardless of replacement level or concrete type. In self-compacting concrete (SCC), RAP replacement at 30%, 60%, 90%, and 100% reduced CS by 19.6%, 32.2%, 40.3%, and 45.9%, respectively [41]. In metakaolin-based geopolymer concrete, even a modest 25% RAP replacement led to a dramatic 42.8% CS reduction [19]. In roller compacted concrete pavement (RCCP), all RAP mixes except 100% total RAP met the minimum CS threshold of 27.6 MPa, though fine RAP consistently yielded lower CS than coarse RAP due to higher asphalt content and gap-graded particle distribution [18]. Conventional concrete with RAP similarly showed a maximum 28-day CS of only ~25 MPa, appreciably lower than the ~28.1 MPa achieved with RCA under the same conditions [26], while a study on structural RAP concrete confirmed consistent CS reductions that scale with replacement ratio across all mix types [24]. Steel fibers added to RAP concrete partially compensated for CS loss; increasing fiber content at 20–60%, RAP replacement raised CS by 2.88–10% over the unfibered RAP baseline [23], and replacing 10% of cement with sugarcane bagasse ash (BGA) in 100% RAP concrete also meaningfully mitigated the CS penalty [29]. At elevated temperatures, the combined use of RAP and RCA in RC beams produced compressive strengths of 21–23 MPa with additive degradation resulting from both aggregate types interacting to weaken the cement matrix [14]. Collectively, these findings, as shown in Table 2, establish a clear hierarchy: untreated RCA and, more markedly, RAP reduce compressive strength relative to natural aggregates, but surface treatment, especially accelerated carbonation and supplementary materials such as fibers, graphene oxide (GO), or pozzolanic additions, can substantially recover or even enhance CS in engineered recycled aggregate concrete systems.

### 6.2. Tensile Strength

The incorporation of RCA and RAP as coarse aggregate replacements consistently reduces the tensile strength of concrete, with the magnitude of the reduction depending on aggregate type, replacement level, and concrete mix system. Abedalqader et al. [13] reported that splitting tensile strength decreased progressively at ambient temperature as RAP replacement increased, with reductions of 8%, 18%, and 28% at 10%, 20%, and 30% RAP replacement, respectively, relative to natural coarse aggregate (NCA) concrete. For RCA, the same study recorded tensile strength reductions ranging from 3% to 14% at 20 °C, widening to 16–44% at 500 °C, while RAP–RCA blended mixes showed combined reductions in which the RAP fraction exerted a more dominant deteriorating effect. The underlying mechanism in both cases is the weakened interfacial transition zone (ITZ) caused by the residual asphalt film coating RAP aggregates and by the old, adhered mortar in RCA, which compromises aggregate–paste bond integrity and promotes premature microcracking under tensile loading [13]. Singh et al. [29] observed a linear relationship (R^2^ > 0.9) between RAP content and splitting tensile strength loss in concrete mixes incorporating coarse RAP (CRAP) and fine RAP (FRAP) aggregates. At full (100%) replacement, CRAP and FRAP reduced splitting tensile strength by 26% and 44%, respectively, at 28 days of moist curing, whereas 50% replacement of either fraction produced equivalent tensile strength to the control, demonstrating that partial replacement can limit strength degradation [29]. Andrew et al. [23] similarly confirmed that split tensile strength decreased monotonically with increasing RAP replacement (20–60%), with the reduction attributed to the weak bond between the cement matrix and the asphalt-coated RAP aggregates, which facilitated easy cracking of the hardened concrete [23]. In ultra-high-performance concrete (UHPC) incorporating 100% fine recycled aggregate (RA) as a complete replacement for natural river sand, Yu and Wu [65] recorded an 8.43% reduction in direct tensile strength (from 7.95 MPa to 7.28 MPa), attributable to increased interfacial transition zone density from numerous RA particle contacts that reduce overall matrix cohesion. In flat slab–column connection specimens, Ferreira et al. [38] identified tensile strength as the concrete property most significantly affected by coarse RCA substitution: slabs reinforced at ρ = 0.7% exhibited approximately 8% tensile strength reduction, while slabs at ρ = 1.2% experienced approximately 30% reduction, with the variation attributed primarily to the inherent quality variability of the recycled aggregate rather than to the replacement ratio itself; notably, the experimental tensile strength exceeded Eurocode 2 lower-bound characteristic values (fctk,0.05) in all tested configurations, as summarized in Table 3. To counteract these tensile strength deficiencies, several mitigation strategies have been investigated. Yu and Wu [65] demonstrated that incorporating graphene oxide (GO) at 0.06 wt% into UHPC with 100% fine RA recovered and exceeded the tensile strength of the natural sand control mix, raising tensile strength to 9.50 MPa, a net gain of 19.50% above the natural aggregate reference by refining pore structure and strengthening interfacial bonding. Andrew et al. [23] established that adding hooked-end steel fibers to RAP concrete progressively improved split tensile strength at all replacement levels and curing ages, with the maximum split strength of 3.1 MPa achieved at 20% RAP with 1.5% steel fibers at 28 days of curing, as the fibers bridged the gap between cement paste and aggregate and resisted crack propagation. Singh et al. [29] found that replacing 10% of ordinary Portland cement (OPC) with sugarcane bagasse ash (BGA) improved the splitting tensile strength of 100% RAP concrete by 13% at 28 days relative to the unmodified 100% RAP mix, with an additional 7% improvement observed at 15% BGA replacement, confirming the pozzolanic densification of the cement matrix as an effective countermeasure to the porosity introduced by RAP aggregates.

### 6.3. Flexural Strength

The incorporation of RCA and RAP as partial or total replacements for natural aggregate consistently reduces the flexural strength of concrete, with the extent of reduction scaling with replacement level and aggregate type, as summarized in Table 4. In metakaolin-based geopolymer concrete, replacing virgin limestone aggregate with 25%, 50%, and 100% RAP progressively reduced flexural strength from a control value of 6.17 MPa by 26.2%, 40.2%, and 42.2%, respectively; the rate of reduction was less severe than the corresponding compressive strength loss, attributed to a shift in fracture plane from through-aggregate splitting in the control to paste aggregate interface debonding in RAP-containing mixes [19]. In roller-compacted concrete pavement (RCCP), regression analysis of 56 mix combinations confirmed that every unit increase in RAP content exerted a dominant negative effect on flexural strength (coefficient −3.20, R^2^ = 0.90), such that mixtures containing above 50% RAP could no longer satisfy the minimum flexural strength threshold, with weak adhesion between the asphalt-coated particles and cement mortar identified as the governing mechanism [48]. In large-scale reinforced concrete (RC) beams, flexural load capacity under ambient conditions was reduced by 3–13.4% across all RAP- and RCA–RAP-replacement schemes relative to the 100% natural aggregate control of 139.1 kN, with the 80% RCA–20% RAP combination sustaining the smallest reduction (3%) and the 20% NA–80% RAP combination the largest (13.4%); under 600 °C exposure, RCA–RAP beams incurred 55–65% flexural capacity loss, and each incremental rise in RAP proportion within the RCA–RAP blends amplified the heated-state reduction by a further 3–10% [66]. A synthesis of experimental data on structural concrete containing RAP confirmed that flexural strength decreases with rising RAP content: at 20% RAP, the own-team-measured reduction was 14% relative to the control, while the asphalt–mortar surface bond rather than the w/c ratio was identified as the variable governing the magnitude of reduction; notably, 20% RAP simultaneously increased flexural toughness by 48% compared with standard concrete [24]. In RC beams cast from 100% RCA, the modulus of rupture was 4.2 MPa versus 4.5 MPa for normal concrete, while crack widths were up to 5% wider and mid-span deflections up to 30% larger, reflecting the reduced aggregate paste bond integrity caused by high RCA porosity and mortar residues on aggregate surfaces [40]. In steel-fiber-reinforced recycled aggregate concrete (SFRRAC) beams, the limit of proportionality (f_L_) of concrete with 20% and 50% coarse RCA was approximately 65% and 60% of the natural aggregate reference value, respectively, without fiber addition [44]. Complete replacement of natural fine aggregate by recycled aggregate (RA) in UHPC reduced the four-point flexural strength from 17.14 MPa (natural sand reference) to 15.94 MPa (−7.0%) due to the proliferation of interfacial transition zones associated with RA and the weakened mechanical properties of adhered old mortar [65]. In UHPC incorporating 100% coarse RCA, flexural strength at 28 days was lower than that of NCA-based UHPC because old mortar in RCA caused highly uneven stress distribution in the tension zone, triggering stress concentrations at microcrack tips within the RCA or at its ITZ under external loading [64]. Similarly, in RA-modified UHPC, peak load decreased progressively with increasing RA substitution rate most severely at 100% RA, and increasing nano-silica (NS) content alone further reduced flexural strength (e.g., from 7.02 MPa to 4.67 MPa at 50% RA, 1% SF, as NS content rose from 0% to 5%), caused by NS agglomeration and conversion to lower-strength calcium carbonate hydrates [67]. To mitigate these reductions, several strategies have been examined. Graphene oxide (GO) at an optimal dose of 0.06 wt% increased the flexural strength of UHPC with 100% fine RA by 23.4% above the RA-only mix (to 19.77 MPa), surpassing the natural sand reference by 15.3%, through accelerated cement hydration and densification of the interfacial transition zone [65]. Surface treatment of RA encompassing physical and chemical modification methods (T1RA through T3RA) restored the flexural strength of treated-RA specimens to near that of the natural aggregate control (21.8 MPa) and, in the highest-performing T2RA group, raised bending ductility indices I5, I10, and I20 to values approximately 1.33–1.69 times higher than the untreated-RA reference [11]. In UHPC with 100% coarse RCA, single addition of 0.5% carbon nanofibers (CNFs) enhanced flexural strength by 63.6%, while 2.0% steel fibers alone increased it by 43.6%, and the hybrid CNF0.25% + SF2.0% system delivered the highest improvement of 95.2%, all relative to the RCA baseline, via simultaneous nano-scale crack arrest and macro-scale crack bridging [64]. In RA-UHPC, increasing steel fiber volume from 0% to 1% and 2% raised flexural strength from 5.87 MPa to 8.4 MPa (+43%) and 15.85 MPa (+170%), respectively, confirming that steel fiber content dominated over nano-silica in governing flexural performance [67]. In RC beams made with 100% RCA, the inclusion of 1% steel fibers by volume increased the average first-crack load by 15–17% relative to beams without fibers through crack bridging, and a modified Eurocode 2 prediction model was validated to accurately capture serviceability-limit-state deflections [40]. In SFRRAC beams, adding 50 kg/m^3^ of steel fibers recovered the limit of proportionality (f_L_) of both 20% and 50% RCA concretes to 97% and 90% of the natural aggregate reference, respectively, with residual post-crack strength governed primarily by fiber dosage rather than by aggregate replacement ratio [44].

### 6.4. Shear Strength

**Figure 2:**
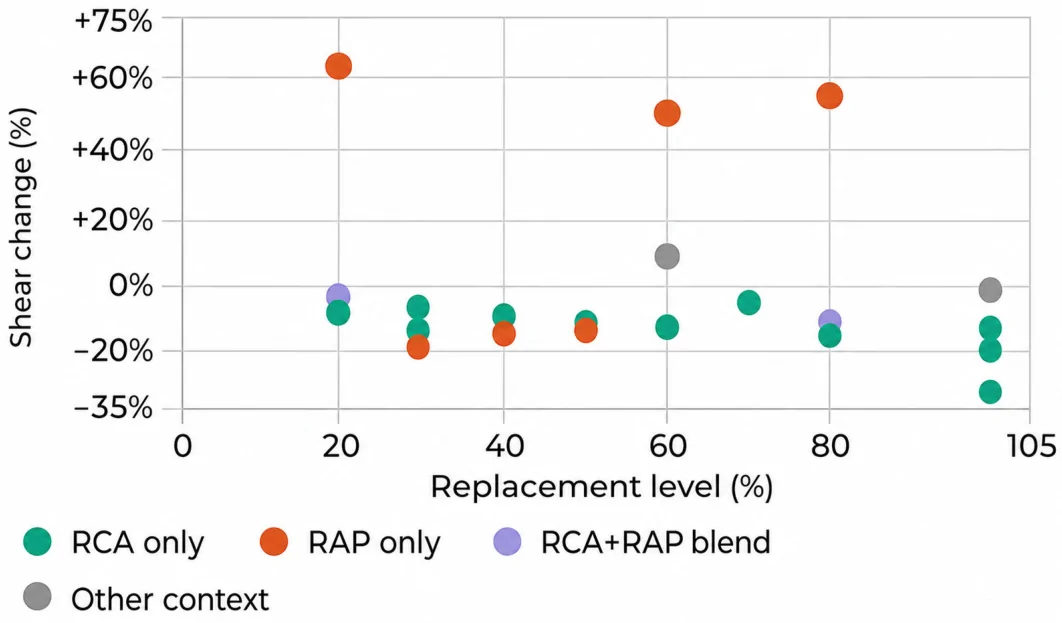
Effect of RCA and RAP replacement level on the shear strength.

The shear strength of reinforced concrete (RC) beams is consistently and notably affected by the incorporation of RCA and/or RAP as aggregate replacements, with the direction and magnitude of the effect depending on aggregate type, replacement level, loading mode, and environmental conditions, as summarized in Table 5 and Figure 2. In a three-group experimental programmer on thirteen large-scale RC beams without stirrups, shear capacity increased as RAP replacement level decreased from 154.2 kN at 100% RAP to 252.1 kN at 20% RAP (a 63% gain) because the rough texture and similar water absorption of RAP to natural aggregate (NA) enhanced aggregate–paste bond; conversely, in the RCA-only group, capacity declined monotonically from 140.2 kN at 20% RCA to 128 kN at 80% RCA due to high absorption, old adhered mortar, microcracks, and deteriorated interfacial transition zone (ITZ) performance, while in the RAP–RCA blended group, RCA content was the dominant factor and increasing RCA reduced shear capacity regardless of RAP presence [27]. Under combined ambient and elevated temperature conditions, RAC-only beams lost approximately 15% of ultimate shear capacity at 400 °C relative to control, while RAP–RAC combination beams suffered significantly greater losses; increasing RAC percentage consistently reduced load-carrying capacity, whereas increasing RAP percentage raised it [14]. At the material level, 100% RCA replacement accelerated shear crack formation, increased longitudinal splitting, reduced shear cracking strength by 14.5% and ultimate shear capacity by 15.6% vs. NA control, and lowered pre-cracking stiffness by 13.68%, all stemming from the lower tensile strength and weaker bonding properties of RCA [49]. Similarly, 100% RCA reduced shear strength of medium-scale RC beams by 12% on average, with RAC beams exhibiting less ductile failure than NA counterparts [56]. In Z push-off specimens, 100% RCA replacement reduced shear strength by 17.3%, with progressive degradation at all five tested replacement levels (25%, 50%, 75%, 100%), driven by reduced fracture toughness and aggregate interlock; a new fracture mechanics-based equation incorporating shear slip and crack opening effects was proposed and validated against an extensive experimental database [34]. For deep beams at 50% replacement, RCA and RAP reduced shear capacity by 9.0%/4.66% and 11.7%/7.52% in the L/D = 1.6 and 2.7 groups, respectively, with RAP causing larger reductions than RCA at equal replacement; when modified mix design with higher cement content was used for 100% replacement, shear capacity of both RCA and RAP deep beams recovered to values nearly equal to NCA control [2]. For full-scale RC beams with stirrups, shear strength decreased by 11–19% at 30% combined coarse and fine RA replacement, by 11% at 100% coarse RA replacement, and by 30% when both fine and coarse RA were fully replaced simultaneously [3]. In steel-fiber-reinforced SCC beams combining RCA with recycled crushed glass (RCG), shear capacity generally decreased with increasing RCA content, except for the 50% RCA + 20% RCG mix, which showed a 5% improvement, while the 100% RCA + 20% RCG mix achieved shear capacity nearly equal to the control, demonstrating a partial compensatory effect of RCG on RCA-induced shear loss [68]. In biaxial shear tests on square RC beams, a 60% RCA + 40% NCA partial blend achieved approximately 8% higher uniaxial shear capacity than both 100% NCA and 100% RCA specimens under all tested load inclination angles [30]. In steel-reinforced RCA (SRRAC) short beams, the RCA replacement ratio (0%, 50%, 100%) had minimal effect on shear bearing capacity, whereas the shear-span ratio was dominant, decreasing from 1.52 to 0.76 and raising shear bearing capacity by 78.1% [57]. Data-driven analyses of large experimental databases confirmed that increasing RCA content consistently and drastically decreased shear strength across 401 collected beam tests, with two ITZs in RCA reducing aggregate interlock through smoother fracture surfaces [32], and tree-model analysis of 334 specimens quantified reductions of 11–19% at 30% RCA, 11% at 100% coarse RCA alone, and 30% at 100% combined fine and coarse RCA, with design code underestimation worsening at higher replacement ratios [69]. In multi-generation recycled aggregate concrete subjected to combined compression-shear loading, shear strength degraded progressively with increasing recycling cycles (n) when normal stress was present, with incremental reductions in contact friction and aggregate interlock identified as the primary mechanisms, driven by the decreasing volume fraction of NCA in Multi-RAC [52]. In two-layer RC beams where rubber recycled aggregate concrete comprised the tension zone (bottom 2/3), FE simulations demonstrated that the higher-grade top concrete layer had no influence on shear resistance, and shear capacity was governed by the lower-grade recycled concrete though shear link-dominated shear resistance, and the impact on overall beam shear capacity was minimal [70]. In semi-precast T-beams with RAC web cores, shear resistance was also governed by the lower concrete grade, while shear links carried the bulk of shear force and precast RAC blocks effectively controlled flexural-shear crack development [71]. Against these reductions, multiple mitigation strategies have demonstrated effectiveness. CO_2_ carbonation treatment of recycled aggregates (CRA) enhanced shear strength relative to untreated RAC beams across all replacement ratios (30–100%), with significant shear reduction only near 50% replacement and a slowing of the decline above 70%; the concrete shear contribution Vc increased with CRA content, bringing CRAC performance closer to NAC [8]. Hooked steel fibers (3D, 4D, 5D) in 100% RCA beams at Vf = 0.25–0.75% progressively restored and exceeded baseline shear capacity 3D at 0.60% Vf, improved shear by 156%, 4D by 136.7%, and 5D at 0.50% Vf by 108.7%, while 0.50% SF nearly fully offset the pre-cracking stiffness degradation induced by 100% RCA [49]. Steel fibers at 1.0% Vf were nearly sufficient to offset shear capacity loss from the combined use of 100% RFA and 100% RCA [72]. Polypropylene fibers at 1% volume were beneficial for shear in beams with up to 30% RCA combined with crumb rubber but proved counterproductive above 30% RCA [61]. The combination of 5% SCBA as cement substitute and 10% RPET as sand substitute produced beams with 17.38% higher shear capacity than conventional concrete, demonstrating that supplementary material synergies can more than compensate for RCA/RAP-related shear deficiencies [73]. CFRP U-wraps increased shear capacity by 36% in NAC beams and 60% in 100% RAC beams, with a higher percentage gain for RAC, confirming that external CFRP strengthening is particularly effective for recycled aggregate beams [46]. CFRP laminates applied to shear-deficient RAC beams likewise produced a higher % shear strength increase for RAC than NAC control beams [74]. GFRP stirrups produced only ~2% difference in shear strength compared to conventional steel stirrups in 100% RCA beams [56]. RCG (20%) used as a fine aggregate substitute partially offset RCA-induced shear reduction in SCC beams, especially at the 50% RCA replacement level [68].

### 6.5. Bond Strength

**Figure 3:**
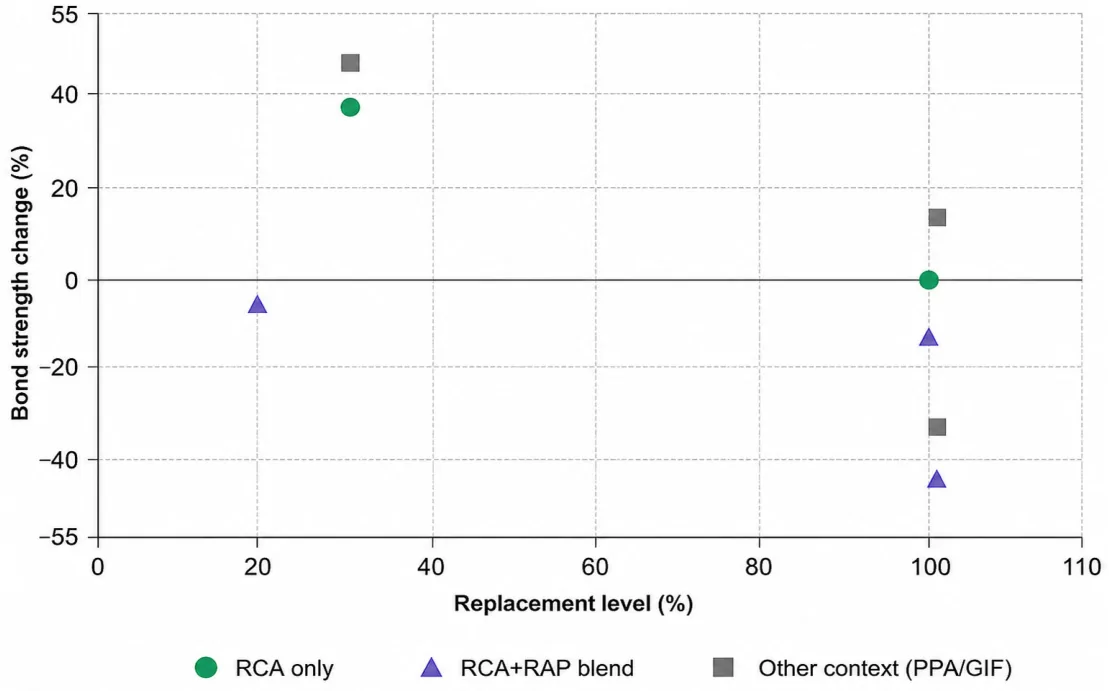
Effect of RCA and RAP replacement levels on the bond strength.

The incorporation of RCA and RAP as coarse aggregate replacements exerts distinct and aggregate-specific effects on the bond strength between steel reinforcement and concrete, with findings from all available studies summarized in Figure 3. In a systematic pull-out programmer using 90 concrete cubes with two bar diameters (10 mm and 12 mm) and fifteen aggregate combinations, the RCA + RAP combination reduced bond stress relative to natural aggregate (NA) samples by 6–15% for the 10 mm bar and by 6–45% for the 12 mm bar, while RAP-only concrete produced the most pronounced reductions in tensile load capacity and bond strength of all tested combinations; conversely, concrete incorporating higher proportions of RCA consistently attained bond values closer to, and in some cases approaching, the NA reference, confirming that RCA is a superior recycled aggregate for bond integrity compared to RAP at equal replacement percentages [45]. In a fundamental mechanism investigation using surface-free energy analysis and pull-off tensile strength tests across three aggregate mineralogizes (limestone, granite, sandstone), three asphalt viscosities, four aging stages, and four mortar grades, the failure mode in RAP-incorporated concrete was governed by the interplay of aggregate mineralogy, asphalt rheology, and aging intensity: granite RAP produced predominantly adhesive failure at the aggregate–asphalt interface; limestone and sandstone RAP at mortar grades ≤ M20 failed cohesively through the asphalt layer, shifting to adhesive failure at the mortar–asphalt interface for higher mortar grades; and as asphalt grade and aging intensity increased, the physical bonding with both aggregate and cement-mortar improved, with Van der Waals interactions identified as the dominant intermolecular mechanism [12]. In a RILEM half-beam bond study on 37 specimens incorporating coarse and fine recycled aggregate (RFA) at multiple water-to-binder ratios and parent concrete strengths, RA did not alter the expected mode of bond failure but significantly weakened bond strength in flexure, with fine RCA degrading bond more severely than coarse RCA and RA sourced from weaker parent concrete producing much inferior interfacial bonding; an almost linear relationship was established between the relative bond strength of RAC and the relative water absorption ratio of the aggregate (for coarse RCA only), a relationship that deteriorated when RFA was also incorporated, indicating that existing NAC design-code anchorage lengths are unsafe for RAC [9] In pull-out tests on RAP concrete before and after thermal shock, initial bond strength decreased with increasing RAP content at ambient temperature (23 °C) due to the soft asphalt coating weakening the concrete matrix and inducing stress concentration; however, at 600 °C, the bond strength reduction was 65.92% for 0% RAP but only 49.46%, 44.28%, and 38.73% for 25%, 50%, and 75% RAP, respectively, demonstrating that higher RAP content protected residual bond under thermal exposure through asphalt flow and crack sealing; additionally, 20 mm bars consistently exhibited 26–61% lower bond strength than 14 mm bars across all temperatures, and rapid water cooling induced greater bond loss than natural air cooling [75]. In eccentric pull-out tests on 100% RCA concrete beams, the bond stress-slip response exhibited a three-phase behavior and varied significantly with the proportion of polypropylene plastic aggregate (PPA) used to partially replace RCA and with beam depth [37]. For the 30% RCA mix, bond strength exceeded both the 0% RCA and 50% RCA mixes by up to 37.3% depending on bar diameter, embedment length, and compressive strength, while the 50% RCA mix reduced compressive strength by 9.8% and split tensile strength by 10.7% relative to natural aggregate concrete in 40 MPa specimens at 28 days; increasing bar diameter from 16 to 20 mm caused a 13.4% average bond reduction across all RCA combinations [76]. To counteract these bond strength deficiencies, targeted improvement strategies have been evaluated. In RCA-based concrete with 100% coarse recycled aggregate, incorporating 5% PPA as a partial RCA substituent increased bond strength by up to 16.6% relative to the RCA-only control, while 10% PPA reduced it by up to 37.5%; the recommended upper limit was established as 5% PPA to achieve net benefit across flexural and bond performance [37]. The addition of galvanized iron fibers (GIFs) at 0.25% and 0.5% by volume to RCA-incorporated concrete increased pull-out bond strength by 40.8% and 46.5%, respectively, relative to no-fiber baselines, with 0.5% GIF in combination with 30% RCA achieving the highest bond performance; the optimal recommendation was established as 0.5% GIF with ≤30% RCA [76]. For the fundamental code design limitation identified in RCA bond, a multiplier λ = 1/(1 − 0.19η − 0.08ξ) was proposed to correct the NAC anchorage length for RAC based on probabilistic analysis of 37 beam specimens, providing a conservative and practically applicable safety factor for RAC structural design [9].

### 6.6. Failure Mode

**Figure 4:**
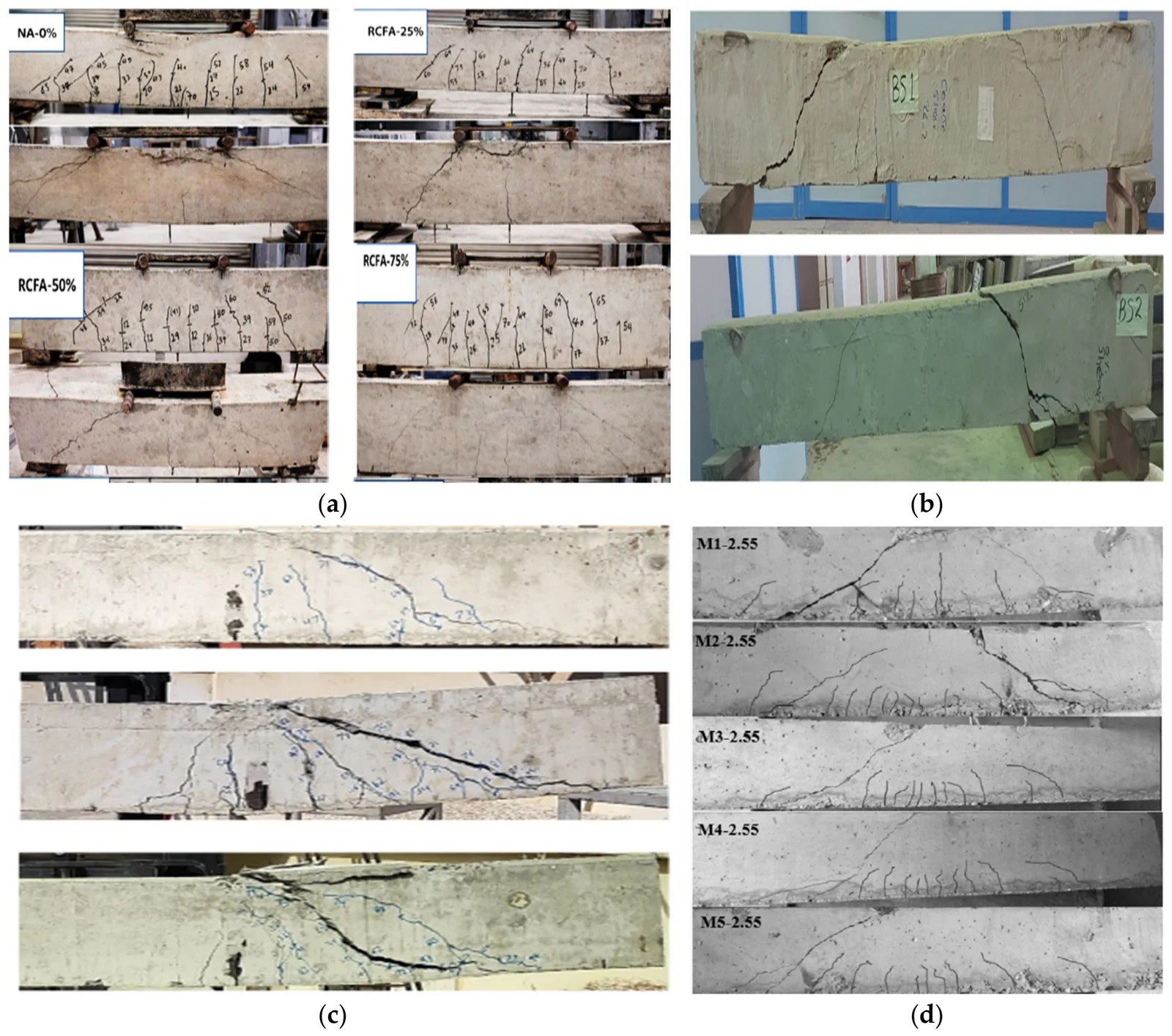
Failure modes of concrete with NA, RCA, and RAP. (**a**) [1], (**b**) [33], (**c**) [56], and (**d**) [68].

The failure mode of reinforced concrete (RC) members made with RCA or RAP is extensively documented across both structural beam tests and material-level fracture investigations, with findings from all 17 available studies summarized in Table 6. In RC beams designed without stirrups under both ambient and elevated temperature conditions, all specimens regardless of whether they incorporated RAC, RAP, or their combinations exhibited an identical brittle diagonal shear failure mode, initiated by mid-span flexural cracking that propagated into a dominant diagonal shear crack; neither the type nor the proportion of recycled aggregate altered this failure pattern, and exposure to 400 °C distributed additional cracks along the beam span without changing the failure mechanism [14]. Under combined compression-shear loading, the failure mode of multi-recycled aggregate concrete (Multi-RAC) shifted from typical shear failure at low normal stress ratios (σ/fc) to complex combined shear-axial collapse as σ/fc increased to 0.8, with this transition governed entirely by the normal stress level rather than by aggregate type or recycling cycle count (n); increasing n progressively flattened the post-peak softening curve, making failure more gradual, while the reduced NCA volume fraction in Multi-RAC decreased aggregate interlock without changing the governing failure mechanism [52]. In Z push-off specimens at RCA replacement levels of 0–100%, Mode II shear fracture governed consistently across all specimens; during initial loading, crack width remained stable then grew rapidly with shear slip after an inflection point at near-ultimate load, and shear transfer strength was reduced by 15% or more when RCA replacement exceeded 30%, with 100% RCA producing a 17.3% reduction [34]. In biaxial shear beam tests on NCA, 100% RCA, and 60% RCA + 40% NCA specimens under load inclinations of 0°, 20°, and 40°, all beams failed by diagonal tension failure in the shear span regardless of aggregate type or load inclination angle, confirming that RCA substitution does not alter the fundamental biaxial shear failure mode [30]. In shear-deficient RAC beams tested alongside NAC controls, the failure modes and crack patterns of unstrengthened specimens were comparable between NAC and RAC, both exhibiting critical diagonal shear cracks at approximately 45° [46]. In RRAC beams exposed to temperatures up to 600 °C, failure modes remained similar to those of reinforced natural aggregate concrete (RNAC) beams at equivalent temperatures, with no explosive spalling occurring during the heating process; elevated temperature degraded shear capacity and initial stiffness but did not alter the fundamental shear failure type [44]. For RC beams with carbonated recycled aggregate (CRA) at 30–100% replacement, the failure mode was consistently shear compression failure with a main diagonal crack connecting the loading point to the support, exhibiting progressively wider crack openings near the loading point; this pattern was identical for CRAC and untreated RAC beams [9]. In shear tests on RAC beams strengthened with CFRP sheets, unstrengthened beams with 20% RCA failed in shear-flexural mode, while the shear crack width increased proportionally with RCA content 5.6 mm at 20% RCA, 6.5 mm at 60%, and 6.9 mm at 100%, compared to 4.9 mm for the NA control, confirming that increasing RCA content promotes more brittle shear behavior [77]. Beams without PP fiber reinforcement containing crumb rubber and RCA failed in a brittle mode along a wide shear crack at ultimate load, with 50% RCA beams consistently exhibiting brittle failure with or without additional crumb rubber [61]. In UHPC containing 50% RCA, the compressive strength was reduced by 2.8% relative to NCA–UHPC, and the post-peak behavior was marginally inferior; exposure to elevated temperatures (200–800 °C) showed that RCA had a positive effect on reducing strength loss [64]. In flexural RC beams made entirely of RCA, the failure mode was not affected by aggregate type; all beams failed in tension-controlled flexure through steel yielding, though RCA beams produced a higher number of closely spaced cracks and a 14.3% lower cracking load compared to natural aggregate beams [26]. The failure mechanism in RAP-incorporated concrete was governed by aggregate mineralogy, asphalt grade, and binder aging: granite RAP produced predominantly adhesive failure at the aggregate–asphalt interface; limestone and sandstone RAP at mortar grades ≤ M20 failed cohesively through the asphalt layer, shifting to adhesive failure at the mortar–asphalt interface for higher mortar grades (>M20); aged asphalt predominantly exhibited cohesive failure; and physical bonding was primarily driven by Van der Waals interactions [13]. In 30% RCA RC beams, the optimum replacement level produced a 24% higher compressive strength than conventional aggregate at 28 days, and unretrofitted RCA beams reached a 57% higher load capacity than conventional concrete beams; load-deflection behavior of RCA beams consistently outperformed conventional controls [78]. To mitigate the more brittle failure modes associated with RCA and RAP, several improvement strategies have been applied. Steel fibers at 1.0% and 1.5% volume fraction in 60% treated-RCA beams fundamentally shifted the failure mode from shear to flexure, confirmed by both crack patterns and load values (196.2 kN and 202.1 kN vs. 166.7 kN for the no-fiber control), while 0.5% SF was insufficient to trigger this transition, producing only a 15% capacity gain without changing the failure type [79]. PP fibers at 1% volume fraction in beams with up to 30% RCA shifted failure from brittle shear along a single wide crack to flexural-shear cracking with multiple distributed diagonal cracks; above 30% RCA, this beneficial transition was not observed [61]. Four continuous CFRP layers on RAC beams reduced shear crack width from 6.9 mm to 0.6 mm and shifted the dominant failure mode toward flexure, fully restoring beam stiffness; two layers produced only partial crack control (1.9–5.1 mm crack widths) without a full mode transition [77]. CFRP U-wrapping changed the failure mode of both NAC and RAC beams from diagonal shear crack failure to CFRP laminate debonding, with RAC beams achieving a 60% shear capacity increase vs. 36% for NAC [47]. CFRP laminate configurations on shear-deficient RAC beams also changed the critical failure mode from diagonal shear crack to FRP debonding; continuous wrapping produced flexural failure in RAC beams without CFRP debonding [74]. Spiral transverse reinforcement combined with full-zone GFRP retrofitting improved ultimate load capacity by 27–60% over reference beams and produced better load-deflection performance, with full-zone GFRP outperforming X-shaped strips by 9–12% [78]. CO_2_ carbonation treatment of RCA maintained the shear compression failure mode while improving shear strength relative to untreated RAC beams, with the failure crack pattern remaining consistent [9]. As depicted in Figure 4, all types of concrete exhibited diagonal brittle shear failure, whereas the cracks were initiated in RCA and RAP specimens earlier than NA specimens.

### 6.7. Elastic Modulus

**Figure 5:**
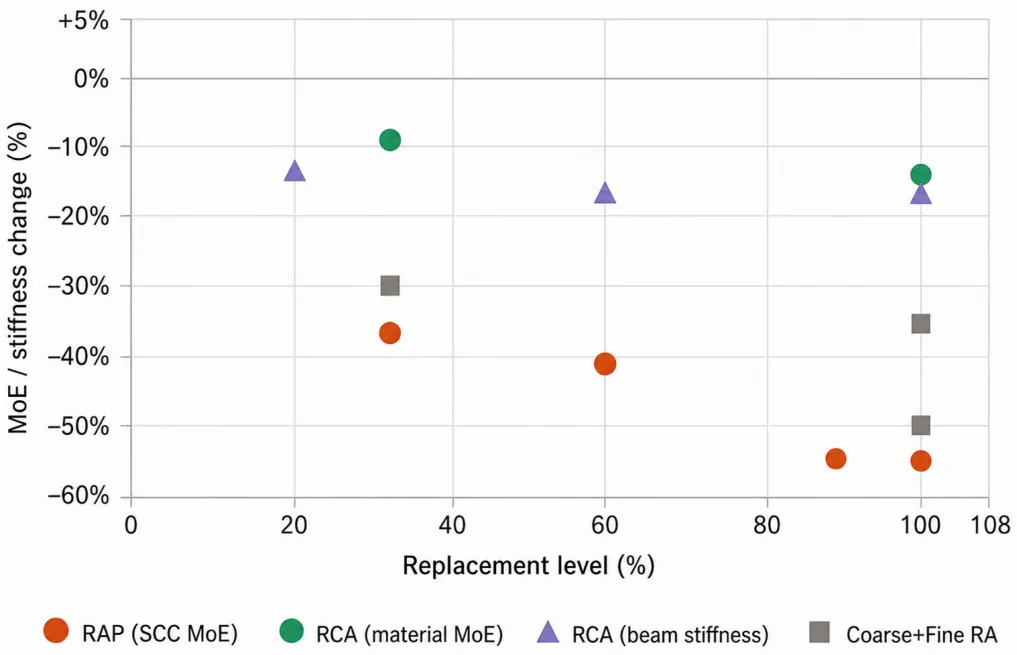
Effect of RCA and RAP replacement level on modulus of elasticity and stiffness.

The modulus of elasticity (MoE) and initial stiffness of concrete and RC members are consistently reduced by the incorporation of RCA and RAP, though the magnitude depends strongly on aggregate type, replacement level, testing conditions, and specimen type, as summarized in Figure 5. For RAP-incorporated self-compacting concrete (SCC), MoE decreased progressively with rising RAP content from 31.759 GPa at 0% to 20.333 GPa at 30%, 18.875 GPa at 60%, 15.012 GPa at 90%, and 14.941 GPa at 100%, representing reductions of 36%, 41%, 53%, and 53%, respectively, while peak strain increased with RAP content, indicating improved energy absorption capacity despite the stiffness loss [42]. For both RAP and RCA, MoE decreased as replacement levels increased at the same temperature and decreased further for a given replacement ratio as temperature rose from 20 °C to 500 °C; the MoE reduction was more sensitive to small RAP replacement percentages but more sensitive to large RCA replacement levels [14]. In full-scale RC beams with coarse and fine recycled aggregates, the static MoE fell from 27.7 GPa (0% replacement) to 19.5 GPa with 30% fine RA only, 19.6 GPa with 30% coarse RA only, and 14.2 GPa when both fine and coarse RA were fully replaced at 100%, a reduction of approximately 49% [4]. In slab–column connection tests at 0%, 30%, and 100% coarse RCA replacement, measured Ec values ranged from 22,420 to 27,470 MPa; the authors noted that variations in Ec were associated with the intrinsic variability of recycled aggregate quality rather than the replacement rate per se, and that all values remained within EC2 theoretical bounds; furthermore, neither pre-cracking nor post-cracking flexural stiffness of the slabs was significantly affected by RCA incorporation [39]. In RC beams without stirrups made with 100% RCA, pre-cracking stiffness was reduced by 13.68% relative to the NA control, an effect attributed to low-strength cement particles and dust on RCA surfaces impairing hydration and bonding at the concrete interfaces [1]. In RRAC beams tested across temperatures from ambient to 600 °C, increasing RCA replacement percentage reduced initial stiffness at every temperature level, with the initial stiffness reduction being more sensitive to elevated temperature than the reduction in load-bearing capacity, because RCA microcracks generated during crushing progressively reduced aggregate toughness [44]. For RC beams with 20%, 60%, and 100% RCA and no CFRP strengthening, structural stiffness values were 10.3, 9.8, and 9.8 kN/mm, respectively, compared to 11.8 kN/mm for the NA reference, corresponding to reductions of 12.7%, 16.9%, and 16.9% [77]. In 100% RCA beams containing waste glass powder, the full substitution of NCA with RCA negatively impacted initial stiffness, ductility, and toughness [33]. Replacing NCA with RCA in SCC beams slightly reduced stiffness, while replacing natural fine aggregate with recycled crushed glass (RCG) partially compensated for this loss [68]. In 30% RCA beams retrofitted with GFRP and spiral transverse reinforcement, all examined beams including those with RCA showed nearly identical stiffness characteristics, with load-deflection performance of RCA beams exceeding that of conventional aggregate controls [78]. In UHPC containing 50% RCA and fine recycled aggregate, MoE was slightly reduced relative to NCA–UHPC, with characteristic properties declining due to multiple ITZs, higher porosity, and weakened adhered mortar [64,65]. Several improvement strategies have demonstrated effectiveness in mitigating RCA/RAP-induced MoE reductions. Carbon nanofibers (CNFs) at 0.25–1 wt% in 50% RCA–UHPC improved MoE by 27–35% above the RCA–UHPC baseline, while steel fibers (SFs) at 0.5–2 wt% increased MoE by 3.5–12.0%; the hybrid combination of CNF and SF further improved RCA–UHPC MoE by 31.0–39.0%, representing the most effective single-material strategy for MoE recovery in UHPC [64]. The addition of GO at 0.02–0.08 wt% to fine-RA UHPC improved MoE by 3.62–12.95%, with the optimal dosage identified as 0.06 wt%, at which GO accelerated cement hydration and refined pore structure sufficiently to recover and exceed the NCA–UHPC elastic modulus [65]. Steel fibers at 0.5% Vf in 100% RCA RC beams nearly fully offset the 13.68% pre-cracking stiffness reduction, with higher fiber volume fractions progressively restoring and improving beam stiffness [1]. Four continuous CFRP layers applied to high-RCA beams (60–100% RCA) restored structural stiffness to levels comparable to the NA reference beam (NC-0-0 = 12.3 kN/mm), while two CFRP layers achieved only partial recovery (11.7 and 11.1 kN/mm for 60% and 100% RCA, respectively) [77]. Waste aluminum fibers (WAFs) at 1% volume fraction improved the initial stiffness, ductility, and toughness of 100% RCA beams, with higher WAF contents of 2% and 3% producing declining returns [34]. RCG at 20% as a fine aggregate substitute in RCA–SCC beams recovered the stiffness losses induced by RCA replacement, with stronger compensatory effects at higher RCG contents [68].

### 6.8. Ductility and Energy Absorption

**Figure 6:**
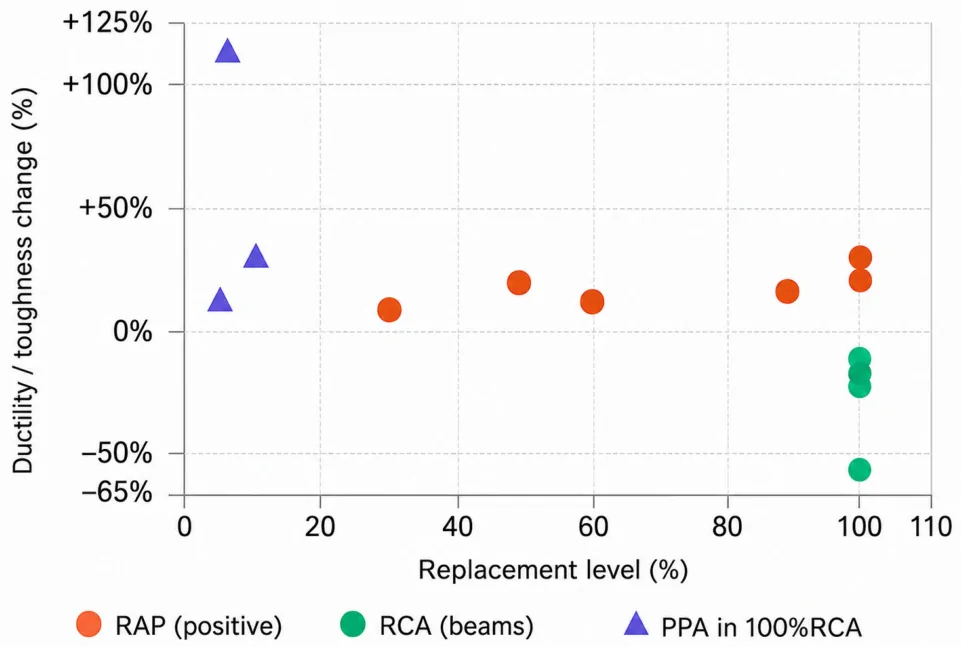
Effect of RCA and RAP replacement level on ductility and energy absorption.

The ductility and energy absorption capacity of concrete and RC members made with RCA and RAP exhibit a strongly aggregate-type-dependent and replacement-level-dependent response, as summarized in Figure 6. For RAP-incorporated self-compacting concrete (SCC), toughness index increased monotonically with RAP content from 1.12 (0%) to 1.21 (30%), 1.25 (60%), 1.31 (90%), and 1.40 (100%), representing progressive improvements of 8.0%, 11.6%, 16.9%, and 25.0%, respectively, while peak strain simultaneously rose from 0.00210 to 0.00299, demonstrating that RAP consistently enhanced SCC’s flexibility and energy absorption capacity despite reducing compressive strength [42]. This beneficial energy absorption trend was confirmed in metakaolin-based geopolymer concrete, where RAP addition at 50% and 100% replacement significantly increased strain at peak compressive strength and produced higher toughness than the virgin-aggregate control, with the better deformation and ductility characteristics suggesting suitability for concrete exposed to impact and cyclic loads [20]. In roller-compacted concrete pavement (RCCP), incorporating up to 10% crumb rubber and 50% RAP was identified as cost-effective and beneficial for prolonging pavement life through increased toughness and energy absorbency, with the optimal combination of 10% crumb rubber and 50% RAP maximizing both metrics [49]. In RC deep beams, RAP-containing specimens absorbed approximately 2–3.7× more energy than both NCA and RCA specimens and exhibited the highest reserved post-peak strength, demonstrating ductility superior to NCA, while RCA deep beams showed the least reserved strength and the most brittle behavior of all three aggregate types [3]. From a fracture mechanics perspective, the apparently contradictory behavior of RAP can be attributed to the residual asphalt film coating the recycled aggregate particles. This compliant bituminous layer weakens the aggregate–cement bond, thereby reducing compressive, tensile, and flexural strengths through premature interfacial debonding. However, the same compliant interface promotes gradual crack propagation, crack deflection, and increased fracture energy dissipation during loading, delaying crack coalescence and enhancing deformation capacity, toughness, and energy absorption. In contrast, the toughening mechanisms associated with RCA are primarily governed by mechanical interlocking and limited crack bridging within the hydrated cement matrix rather than by interfacial flexibility. Consequently, RAP generally exhibits a greater toughening effect than RCA at low-to-moderate replacement levels, where the increase in fracture energy outweighs the reduction in bond strength. At higher RAP replacement ratios, however, excessive interfacial weakening becomes dominant, leading to an overall deterioration in mechanical performance despite the continued improvement in deformation capacity. In contrast to RAP’s ductility-enhancing effects, RCA consistently reduced ductility in structural applications: 100% RCA in RC flexural beams decreased ductility and increased crack severity through accelerated crack formation and longitudinal splitting [1], and full substitution of NCA with RCA in beam specimens negatively affected ductility and toughness alongside initial stiffness [34]. In RC flexural beams, 100% RCA decreased beam ductility by 15.9% and TWW by a further 8.7%, both relative to fresh-water, granite-aggregate reference beam [26]. In semi-precast T-beams with 100% RAC web cores, displacement ductility ratios were 11.6 (high-strength RAC, −10% vs. reference NAC = 12.9), 5.8 (low-strength RAC, −55%), and 8.0 for precast RAC blocks, confirming that RAC grade is a critical design parameter governing ductility in hybrid beam systems [71]. In RA–UHPC without steel fiber reinforcement, specimens exhibited a linear-elastic load-deflection response followed by a sharp post-peak drop with negligible residual strength quasi-brittle behavior, and increasing RA substitution rate slightly decreased toughness [67]. For UHPC with 100% recycled fine aggregate (RFA), tenacity (fF/fc) decreased progressively as more RFA was introduced, driven by the growing total length of ITZs and their degraded quality under standard curing conditions [50]. In 100% RCA RC beams with 100% RCA and PPA as a partial substitution for RCA, the deflection capacity increased by up to 30% with increasing PPA content, and ductility and toughness improved by up to 11.7% and 113.6%, respectively, depending on PPA content and beam depth [38]. In RPET–SCBA beams (10% RPET as coarse aggregate substitute and 5% SCBA as cement substitute), flexural capacity was reduced by 11% vs. conventional beams, though crack patterns and ductility remained comparable [73]. In 30% RCA beams with spiral reinforcement, load-deflection performance exceeded that of conventional aggregate beams, and spiral reinforcement improved ductility potential by constraining the concrete core [78]. Several improvement strategies have been demonstrated to effectively restore or enhance ductility and energy absorption. Steel fibers in RA–UHPC shifted the load-deflection response from quasi-brittle (sharp post-peak drop) to ductile (three-phase response with smoother descent), with increasing SF content progressively improving toughness and residual strength via bridging and crack-growth resistance mechanisms [67]. Autoclaved curing of RFA–UHPC significantly enhanced tenacity beyond standard-curing levels by improving ITZ microhardness and reducing ITZ thickness, recovering the fF/fc ratio at all RFA replacement ratios [50]. Hooked steel fibers (3D/4D/5D) at 0.5% V_f_ offset the pre-cracking stiffness reduction of 100% RCA beams and enhanced ductility through delayed crack initiation and improved post-cracking response [1]. Steel fibers at 20–50 kg/m^3^ in RC–SFRRAC beams improved ductility more significantly than the RCA substitution ratio itself and at sufficient content could partially or fully replace conventional rebar reinforcement while maintaining ductility [45]. PP fibers at 1% volume fraction restricted crack propagation in RCA + CR beams and increased deformation and energy absorption capacity, with the optimal combination of 30% RCA + 5% CR + 1% PP achieving the best resistance and deformability [61]. SFRC jacketing with 2% SF substantially increased the maximum flexural strength and displacement of 100% CRA RC beams when applied both before and after failure, with ductility significantly improved relative to unjacketed CRA beams [80]. Fly ash (FA) at 20% OPC replacement increased concrete maximum compressive strain and negligibly increased ductility of RCA beams, partially offsetting the 15.9% ductility reduction induced by 100% RCA [26]. Waste aluminum fibers (WAFs) at the optimal dosage of 1% volume fraction significantly improved ductility and toughness of 100% RCA beams, with higher dosages showing diminishing benefit [34]. Precast RAC block construction in hybrid T-beams recovered displacement ductility ratio from 5.8 (cast in situ low-strength RAC) to 8.0 and was highly effective in controlling flexural-shear crack development [71].

## 7. Effect of RCA and RAP on Concrete’s Physical and Durability Properties

### 7.1. Workability

The incorporation of RCA and RAP as aggregate replacements consistently reduces the workability of fresh concrete across conventional, self-compacting, and ultra-high-performance concrete systems, with the direction and magnitude of the effect governed by aggregate type, surface condition, replacement level, and particle gradation, as summarized in Table 7. For RAP-incorporated self-compacting concrete (SCC), workability declined with increasing RAP content, slump flow ranged from 680 mm at low RAP levels to 510 mm at 100% RAP, while the T500 flow time rose from 3.2 s to 7.6 s, yet all mixes remained within SCC specification bounds [42]. In conventional concrete, RAP consistently reduced slump due to the asphalt mortar coating, dirt particles, and irregular aggregate shape; at 20% RAP, workability declined further as steel fiber (SF) content was increased from 0% to 1.5%, reaching minimum slump at 1.5% SF, though the resulting mix remained viable for practical construction use [24]. In RAP concrete incorporating coarse and fine fractions, the aggregate type was critical: the NA control achieved a slump of 15.5 mm, 100% coarse RAP (CRAP) reduced it to 11 mm (a 29% reduction), while complete replacement with fine RAP (FRAP) caused 100% workability loss; zero slump attributed to the high viscosity of the asphalt film and the substantially greater surface area and angularity of fine RAP particles [30]. For RCA in self-compacting concrete, the control mix achieved a maximum slump flow of 695 mm (T500 = 2.4 s); replacing NCA with 50% RCA and 100% RCA reduced slump flow to 640–655 mm and 600–625 mm, respectively, driven by old mortar on RCA surfaces absorbing free water and leaving the mix dry; V-funnel time increased slightly, while L-box blocking ratio reduction was negligible, confirming adequate passing ability across all mixes [68]. In UHPC, direct addition of untreated RCA reduced flowability and setting time due to high water absorption and surface roughness of RCA particles, while untreated RA (URA) further reduced workability through irregular cracks and voids that absorbed free water and through frictional resistance between angularly shaped particles [12,48]. Several improvement strategies have been demonstrated to effectively mitigate workability reductions caused by RCA and RAP. CO_2_ carbonation treatment of RCA lowered the aggregate’s water absorption, which increased the flowability and setting time of CRCA–UHPC back toward, and in some cases beyond, the NA–UHPC baseline; the resulting CRCA–UHPC also achieved 9.1% higher compressive strength than natural aggregate UHPC [48]. Surface modification of RA through acid washing (T1), cement slurry coating (T2), or chemical strengthening (T3) improved the apparent density and reduced water absorption of RA particles, yielding approximately 25% improvement in slump flow and collapse flow relative to URA–UHPC specimens; chemical strengthening further formed a hydrophobic film on the aggregate surface that helped maintain fresh properties over time [12]. The use of recycled crushed glass (RCG) as a partial fine aggregate substitute (10–20%) in RCA–SCC mixes partially recovered workability losses induced by RCA; mixes with 20% RCG consistently exhibited approximately 15–25 mm higher slump flow than equivalent mixes with only 10% RCG at both 50% and 100% RCA levels, attributed to the lower water absorption and smoother surface texture of glass particles compared to natural fine aggregate [68]. For RAP concrete with bagasse ash (BGA) as a 10–15% cement substitute, higher superplasticizer (SP) dosages were required for 100% RAP, 10% BGA, and 15% BGA mixes to achieve target workability, as BGA’s hygroscopic nature increased water demand; the strategy nonetheless improved hardened mechanical and durability properties of 100% RAP concrete [30].

### 7.2. Water Absorption

The water absorption and porosity of concrete incorporating recycled aggregates depend critically on aggregate type, surface condition, and pore structure. Characterization of five Italian RAP sources revealed that coarse RAP (10 mm) water absorption by immersion (0.81–1.32%) falls within the natural aggregate (NA) range and is substantially lower than that of RCA (3.5–7.91%); however, MIP open porosity of all five RAP types (3.3–6.5%) exceeded the NA average of 2.7%, with the most porous sources (BO = 6.5%, PI = 6.1%) exhibiting inferior freeze–thaw resistance (class F2/FEC4 vs. F1/FEC2 for the remaining three sources) [23]. The hydrophobic bituminous coating (contact angle 130–135° vs. ~40° for NA granite) impeded water penetration, requiring 48 h to reach SSD condition and necessitating under-vacuum testing to capture true accessible porosity—while also causing drying shrinkage of 100% RAP concrete (0.043–0.114%) to exceed the NA reference (0.039%), with BO being the only source surpassing the EN 12620 structural limit of 0.075% [23]. In UHPC with 50% silicomanganese slag (SS) as recycled sand, the porous untreated SS surface absorbed excess water from the mix, leaving insufficient water for hydration, producing a 5 nm ITZ, 133 MPa compressive strength, and 3.10% freeze–thaw mass loss after 800 cycles [11]. Water glass (sodium silicate) immersion at the optimal 2% concentration filled SS surface pores, reduced aggregate water absorption, and densified the ITZ to 3 nm, increasing compressive strength to 158 MPa and reducing freeze–thaw mass loss to 2.69%; over-treatment at 8% wrapped the aggregate surface, delaying hydration and worsening ITZ to 9.5 nm, reducing strength to 131 MPa, and raising mass loss to 3.45% [11].

### 7.3. Porosity

The porosity of concrete and UHPC incorporating recycled aggregates or recycled powder is consistently affected by aggregate type, replacement level, and the intrinsic pore structure of the recycled material, as summarized in Table 8. In UHPC where recycled concrete powder (RCP) or recycled paste powder (RPP) replaced cement, water-permeable porosity increased progressively with replacement rate: RCP at 10%, 30%, 50%, and 70% of cement raised porosity by 27.1%, 68.8%, 92.0%, and 148.2%, respectively, while RPP caused larger increases of 37.6%, 100.1%, 181.0%, and 286.4%; when used as silica fume (SF) replacement, RCP raised porosity by 12.0–73.4% and RPP by 27.8–99.4% at the same ratios, with RCP consistently producing a finer pore structure (MIP paste porosity: SF-70RCP = 9.52% vs. SF-70RPP = 13.40% vs. control = 1.88%) due to its better microaggregate filling effect [63]. In UHPC incorporating recycled fine aggregate (RFA) at 0–100% as natural fine aggregate replacement, the inherently higher porosity of RFA (attributable to the old cement matrix) directly weakened overall matrix density; more RFA increased total ITZ length and area (parameters w_1_ and w_2_), while the comprehensive microstructural parameter w_3_ decreased, confirming progressive weakening of the average microhardness of all material phases, with mechanical properties degrading almost linearly with increasing ITZ content [50]. For RAP aggregate, open porosity (MIP) of five Italian RAP sources ranged from 3.3% to 6.5%, consistently exceeding the natural aggregate average of 2.7%, with microporosity as a noteworthy contributor; the hydrophobic bituminous coating (contact angle 130–135° vs. ~40° for granite NA) impeded water penetration, requiring 48 h for SSD condition and demanding under-vacuum testing to capture true accessible porosity, while the most porous sources (BO = 6.5%, PI = 6.1%) exhibited the poorest freeze–thaw resistance (class F2/FEC4) [23]. In roller-compacted concrete pavement (RCCP), however, RAP inclusions reduced rather than increased total permeable voids relative to the NA control at both 28 and 91 days: at 28 days, 100% coarse RAP reduced TPV by 29%, 100% fine RAP by 6%, and 100% combined RAP by 38% because the asphalt film on coarse RAP melted during the boiling test and infiltrated the capillary void network; the authors concluded that existing standard methods for porosity and water absorption determination are not valid for RAP-inclusive specimens [19]. To mitigate RFA-induced porosity degradation in UHPC, autoclaved curing (190–200 °C, 1.2 MPa) reduced ITZ thickness and enhanced microhardness of both old and new cement matrix and ITZ phases: the dominant mechanism for strength recovery with ITZ quality rather than thickness identified as the key factor governing UHPC performance [50].

### 7.4. Freezing and Thawing Resistance

The freeze–thaw resistance of concrete and UHPC incorporating recycled aggregates is governed by aggregate porosity, microstructure compactness, and the quality of the interfacial transition zone, as summarized in Table 9. In UHPGC incorporating 100% metallurgical-slag RFA (905 kg/m^3^) as the sole fine aggregate, all specimens showed good freeze–thaw resistance after 300 rapid freeze–thaw cycles (−20 °C to +20 °C), with compressive strength loss and mass loss both remaining low across all mixes; this is attributed to the compact microstructure with extremely low porosity formed in the UHPGC matrix and the good ITZ compatibility between the geopolymer paste and RFA [60]. In UHPC where 100% fine recycled aggregate (fine RA) replaced natural fine aggregate, freeze–thaw resistance was marginally deteriorated relative to natural sand: after 300 cycles, the remaining mass of the fine-RA mix (U0) was 99.22% vs. 99.37% for the natural sand control (UR), and the relative dynamic elastic modulus was 95.93% vs. 96.54% reductions driven by the higher porosity and weakened ITZ properties of the adhered old mortar in fine RA [65]. For RAP aggregate, freeze–thaw mass loss over 10 cycles (EN 1367-1 in water and EN 1367-6 in NaCl) varied directly with source porosity: AN, AR, and MA with lower MIP open porosity achieved mass losses of 0.89–0.96% (water) and 0.88–0.97% (NaCl), classified F1/FEC2 (good); BO and PI, the most porous sources (6.5% and 6.1%), recorded mass losses of 1.63% and 1.96% in water and 2.25% and 3.27% in NaCl, classified F2/FEC4 (poor), with post-freeze–thaw water absorption increasing progressively as the bituminous layer was partially removed by cycling [23]. To mitigate the marginal deterioration in freeze–thaw resistance induced by fine RA in UHPC, GO at 0.06 wt% (optimal dosage) increased remaining mass to 99.87% and relative dynamic elastic modulus to 98.51% after 300 cycles, both surpassing the natural sand reference (UR) by refining the pore structure and improving cement hydration [65]. In UHPGC, steel fiber at increasing volume fractions reduced compressive strength loss: the 3 vol% fiber mix (UHPGC-S3) achieved 10.69% lower strength loss than the no-fiber control (UHPGC-S0) after 300 cycles by bridging microcracks and inhibiting their propagation; additionally, optimizing the precursor component by increasing RHA content (UHPGC-R2, 135 kg/m^3^ RHA) produced the lowest overall compressive strength loss of 5.82% through deeper reaction degree and pore structure refinement [60].

## 8. Mechanism-Targeted Effectiveness of Enhancement Strategies

Enhancement strategies reported throughout this review target the distinct failure mechanisms of RCA and RAP differently, and their effectiveness is not interchangeable between the two materials. Strategies that chemically modify or densify a porous, cementitious aggregate surface such as CO_2_ carbonation treatment, water glass (sodium silicate) immersion, acid washing, cement slurry coating, and graphene oxide addition operate by filling and strengthening a porous old-mortar layer, which is the mechanism underlying ITZ degradation in RCA. Carbonation treatment raised CRCA–UHPC compressive strength by 9.1% above the natural aggregate baseline and improved shear performance across 30–100% RCA replacement, while water glass immersion densified the ITZ from 5 nm to 3 nm and increased compressive strength from 133 to 158 MPa within an optimal 2% concentration window, with over-treatment at 8% reversing this benefit. None of the reviewed studies report applying carbonation or water-glass treatment directly to RAP aggregate, and no mechanism for such treatments exists on a hydrophobic, non-porous bitumen coating, so these strategies should not be assumed to transfer to RAP-based mixes. By contrast, strategies that bypass the aggregate–paste interface rather than repairing it are effective for both materials because they do not depend on the chemical nature of the weak bond. Hooked-end steel fibers act through crack bridging rather than adhesion repair, which is why fiber addition improved RAP concrete’s split tensile strength progressively at all replacement levels (maximum 3.1 MPa at 20% RAP with 1.5% steel fibers) and also restored RCA shear capacity by up to 156% at 0.60% fiber volume for 3D hooked fibers. Supplementary cementitious materials such as bagasse ash densify the surrounding bulk paste rather than the aggregate surface itself, improving 100% RAP concrete’s splitting tensile strength by 13–20% despite leaving the bitumen coating chemically unaltered. External FRP strengthening bypasses the internal bond mechanism entirely: CFRP U-wraps produced a larger percentage shear capacity gain in RAC beams (60%) than in NAC beams (36%), consistent with external confinement compensating most where the internal aggregate–paste bond is weakest. The distinguishing principle is therefore mechanistic rather than material-specific: treatments that depend on modifying a cementitious surface are RCA-specific by mechanism, while treatments that mechanically or externally bypass the aggregate–paste interface are effective for RCA and RAP alike.

## 9. Sustainability and Environmental Impact

Using RCA and RAP in new asphalt and concrete mixtures provides a sustainable alternative solution to the construction sector challenges like resource depletion, carbon emissions, and energy demand. These recycled materials contribute directly to achieving sustainability and circular economy goals. Sustainability refers to utilizing natural resources without impacting the demand of future generations. Therefore, using RAP instead of natural aggregates conserves the aggregates for future needs [14,46,82].

### 9.1. CO_2_ Reduction and Environmental Impact

The incorporation of recycled aggregates and recycled powders into cement-based systems consistently yields measurable reductions in CO_2_ emissions and broader environmental impacts relative to conventional production, as summarized in Table 10. In UHPC, replacing 20% of sand with carbonated recycled fine aggregate (RFA) enabled a 5% reduction in Global Warming Potential (GWP) evaluated by life cycle assessment while simultaneously reducing UHPC cost by more than 15%; the pre-carbonation treatment embeds atmospheric CO_2_ into the RFA matrix, thereby contributing to carbon sequestration as well as performance improvement [8]. Substituting recycled concrete powder (RCP) for cement in UHP–ECC produced more substantial CO_2_ savings, as RCP carries a carbon footprint of only 0.001 kg CO_2_/kg compared to 0.83 kg CO_2_/kg for cement; at 75% cement replacement (C75), the carbon footprint decreased by 61.7% vs. the reference mix, and the combined composite substitution of 50% cement + 100% GGBS + 100% silica sand with RCP (C50G100S100) reduced carbon footprint by 48.3% [83]. At the pavement system level, EIO-LCA of RAP-incorporated Portland cement concrete (RAP–PCC) pavement quantified environmental reductions across multiple impact categories vs. plain PCC: petroleum-based fuel use −13.86%, CO fossil −0.72%, NOx −3.13%, PM10 −11.52%, water withdrawals −6.70%, and human health non-cancer impacts −37.87% for single-lift RAP–PCC, with the two-lift configuration (RAP in the bottom lift) achieving greater social and environmental benefits [5]. Despite these environmental benefits, a production-chain cost analysis of RAP aggregate for structural concrete in Italy revealed that current RAP unit cost is 155.39% higher than natural aggregate, primarily due to asphalt milling, recycling fees, and transportation distance though the transformation and assessment phases of RAP production are 41.91% and 31.80% cheaper than the equivalent NA phases, indicating that targeted optimization of the critical production operations could render RAP cost-competitive [18].

### 9.2. Life Cycle Assessment (LCA)

Life cycle assessment (LCA) of RCA and RAP in concrete and pavement systems consistently demonstrates environmental savings beyond what GWP metrics alone capture, as summarized in Table 11. For multi-generation recycled aggregate concrete (MGRAC), LCA quantified GWP values of 358.65, 353.82, 353.75, and 353.67 kg CO_2_-eq/m^3^ for virgin aggregate concrete (VA) and recycling generations RA-1, RA-2, and RA-3, respectively, representing minor but progressive reductions with each recycling cycle; critically, each generation diverts 1060 kg/m^3^ of virgin aggregate from extraction and keeps waste concrete out of landfill for at least three recycling generations, representing a significant avoided-burden benefit not fully captured in GWP [84]. A comprehensive EIO-LCA of RAP–PCC pavement systems demonstrated that single-lift RAP–PCC yielded the highest economic benefit (−10.90% total economic impact vs. plain PCC), while the two-lift RAP–PCC configuration with RAP aggregate in the bottom lift achieved the greatest combined social and environmental performance, including energy use reductions of −4.13%, water withdrawals of −9.20%, smog −6.21%, and a net greenhouse gas reduction of −1.30%; the two-lift design is therefore recommended when environmental and social priorities outweigh economic savings [5,85,86]. A systematic review of RAP in rigid pavement construction identified that while RAP use inherently reduces landfill disposal burden and supports circular economy objectives, comprehensive and independent LCA data for RAP–PCC systems remain scarce; this review highlights the need to generate rigorous LCA studies and to quantify circular economy benefits at the system level rather than the material level, particularly for structural concrete applications where RAP content and processing method vary widely [6,87].

### 9.3. Cost-Effectiveness and Economic Savings

The cost-effectiveness of incorporating RAP into concrete depends critically on aggregate fraction, replacement level, processing strategy, and the local availability of RAP as a waste by-product, with findings across all reviewed studies summarized in Table 12. In experimental concrete mixes with RAP as coarse and fine aggregate replacement, maximum cost savings of 6.13% were achieved at 60% RAP replacement and 1.64% at 20% RAP replacement relative to the NA control mix, with RAP treated as a near-zero material cost input (transport cost only); the use of RAP was identified as a long-term solution for reducing construction material costs and landfill disposal expenses [24,86]. Incorporating RAP aggregates blended with 10% bagasse ash (BGA) as a partial cement substitute reduced the total cost of 1 m^3^ of concrete by more than 40% compared to conventional concrete, with both RAP and BGA available as local waste materials at negligible raw cost; 10% BGA was identified as the optimum replacement for simultaneously enhancing mechanical and durability properties while maximizing economic benefit [30]. An EIO-LCA cost analysis of RAP–PCC pavement showed that aggregate cost comprises 20–30% of total concrete pavement material cost, and single-lift RAP–PCC reduced total economic impact by 10.90% vs. plain PCC; RAP haulage ($155,171) was substantially lower than virgin aggregate haulage ($379,333 for plain PCC) due to shorter transport distance and local stockpile availability, and landfill diversion of waste RAP generated additional savings of $17,734 per pavement unit [5]. A production-chain cost analysis of RAP aggregate for structural concrete in Italy found that RAP is currently 155.39% more expensive than NA when the full production chain including asphalt milling (P1.R), end-of-waste certification (P2.R), and transport (P5.R) is accounted for; however, simultaneously optimizing all three critical operations would reduce RAP cost by 39.64% vs. NA and by 45.13–67.30% vs. RCA, making it the most competitive recycled aggregate for structural concrete applications [18].

### 9.4. Statistical Heterogeneity and Synthesis Limitations

The magnitude and variability of the mechanical property changes summarized in this review reflect substantial methodological heterogeneity across the underlying studies rather than a single, well-defined dose–response relationship. Reported reductions in compressive strength ranged from negligible to as much as 26% for full RCA replacement, while shear capacity reductions clustered more narrowly between 9% and 17.3% across independent studies using comparable replacement levels, indicating moderate consistency for this indicator relative to others. This variability stems primarily from differences in replacement ratio, parent concrete strength grade, water-to-binder ratio, curing regime, aggregate source and processing method, and the testing standard applied across studies, none of which were held constant across the reviewed literature. Because these studies report outcome metrics in non-uniform formats and rarely provide sufficient raw data (sample size, standard deviation) for valid statistical pooling, a formal random-effects meta-analysis with forest plots and I^2^ heterogeneity statistics was not undertaken; attempting to pool such heterogeneous effect measures would risk producing a statistically precise but practically meaningless composite estimate. Similarly, a funnel-plot assessment of publication bias was not performed, as the diversity of outcome metrics and replacement ratios across studies precludes a single comparable effect-size axis. These are acknowledged as limitations of the present synthesis, and future work compiling raw primary data across a narrower, more homogeneous subset of studies (e.g., compressive strength at a fixed 100% RCA replacement level) would be better positioned to support formal meta-analytic pooling.

## 10. Conclusions

This review has systematically compared the mechanical, physical, microstructural, durability and environmental characteristics of recycled concrete aggregate (RCA) and reclaimed asphalt pavement (RAP) as construction materials, drawing upon the peer-reviewed literature published between 2010 and 2026. The principal conclusions are summarized as follows:

- Full RCA replacement can reduce compressive strength by up to 26%, while splitting tensile strength reductions range from 3 to 14% at 20% replacement and reach 26% (CRAP) to higher magnitudes for full FRAP substitution; the magnitude scales consistently with replacement ratio and is governed by ITZ degradation rather than aggregate strength loss alone.
- Combined RCA–RAP beams incurred 55–65% flexural capacity loss relative to conventional concrete, substantially exceeding the loss observed for either material individually, indicating a non-additive interaction between the two ITZ degradation mechanisms rather than a simple superposition of effects.
- 100% RCA replacement reduced shear cracking strength by 14.5% and ultimate shear capacity by comparable margins across independent studies (12–17.3%), while 100% RAP–RCA combinations produced shear-axial interactions not observed in either single-material system.
- Fiber reinforcement reversed these losses at specific dosages: hooked-end steel fibers at 0.60% Vf restored and exceeded baseline shear capacity by 156% (3D fibers) and 136.7% (4D fibers), while CFRP U-wraps increased shear capacity by 36% in RCA-deficient beams—demonstrating that mechanical penalties from recycled aggregate are recoverable through targeted, quantifiable interventions rather than requiring replacement-ratio limits alone.
- Bond strength with reinforcement is replacement-ratio non-monotonic: 30% RCA mixes exceeded both 0% and 50% RCA bond strength by up to 37.3%, indicating an optimum RCA fraction exists rather than a uniform degradation trend, a finding with direct design-code implications for minimum cover and development length provisions.
- Environmental benefits are substantial but application-dependent: RCP substitution for cement reduced carbon footprint by up to 61.7% at 75% replacement, while RAP–PCC pavement systems achieved 10.90% lower total economic impact and human health non-cancer impact reductions of 37.87% versus plain PCC—benefits that are structurally distinct from, and should not be conflated with, the mechanical performance trade-offs above.
- Machine learning models (XGBoost, gradient boosting) predicted shear and bond strength of RAC beams with accuracies exceeding 95%, suggesting these tools are approaching design-ready reliability for recycled aggregate systems where empirical formulas remain limited.

In summary, RCA is better suited for structural concrete applications where quality control and mix design optimization can be applied, while RAP demonstrates superior efficiency in asphalt mixtures, stabilized base layers, and pavement recycling contexts. When used within appropriate replacement limits and with the right enhancement strategies, both materials represent viable and sustainable alternatives to natural aggregates in modern construction.

## 11. Research Gaps and Future Directions

Despite the extensive body of literature reviewed herein, several significant gaps and unresolved challenges remain, which warrant further investigation:

- The long-term durability performance of structural elements made with high-content RCA or RAP under real service conditions remains insufficiently characterized. Most experimental studies are limited to short-term mechanical testing under controlled laboratory conditions, with few investigations examining multi-decade performance, creep, shrinkage evolution, and fatigue under cyclic loading.
- The behavior of combined RCA and RAP in concrete mixes is an emerging area that has received limited systematic study. The interaction between asphalt-coated particles and mortar-coated aggregates within the same matrix may introduce complex ITZ configurations that differ fundamentally from those observed in single-material replacement scenarios.
- The influence of source variability on material consistency presents a major challenge to standardization. RCA quality varies significantly depending on the source concrete strength, demolition method, and degree of mortar removal. Similarly, RAP characteristics are highly influenced by pavement age, traffic loading, binder grade, and milling technique. Developing robust quality control protocols and standardized characterization methods for both materials is critical for broader adoption in structural applications.
- The development of treatment and enhancement methods such as accelerated carbonation, surface washing, polymer impregnation, and silane treatment tailored specifically for use with RCA and RAP in combination offers fertile ground for future research. Such methods, if optimized, could unlock the potential of these recycled materials for structural applications while meeting sustainability targets.

## Figures and Tables

**Table 1:** Comparative physical and mechanical properties of RCA and RAP.

References	Property	Natural Aggregate (NA)	RCA (Typical Range)	RAP (Typical Range)	RCA vs. NA (Change)	RAP vs. NA (Change)
[2,13,14,20,26,27,28,29,30]	Specific Gravity (SSD)	2.55–2.70	2.16–2.53	2.20–2.49	↓ 2–15% due to porous mortar	↓ 5–17% due to lower-density bitumen-coated particles
[25,31,32,33,34,35,36,37]	Apparent Specific Gravity	2.60–2.75	2.43–2.71	2.21–2.59	↓ 3–8%	↓ 6–18%
[29,35,38,39]	Bulk Density—Loose (kg/m^3^)	1450–1650	1297–1500	1365–1428	↓ 5–15% mortar lowers unit weight	↓ 3–12% bitumen reduces particle mass
[24,28,31,34,40]	Bulk Density—Compacted (kg/m^3^)	1600–1750	1397–1600	1820–2300 (Mg/m^3^ range: 1.94–2.30)	↓ 5–12%	±similar to ↑ slightly bitumen acts as lubricant improving compaction
[2,6,8,9,13,14,20,26,27,28,29,30,34,35,40,41,42]	Water Absorption (%)	0.28–2.00	1.50–9.00	0.20–3.50	↑ 3–5× NA porous adhered mortar	↑ slight–2× NA bitumen seals pores but impedes saturation
[8,18,19,22,24,43,44]	Total Open Porosity (%)	1.5–4.0	3.0–15.0	2.0–8.0	↑ high microcracks in residual mortar	↑ moderate porosity sealed by bitumen in standard tests
[13,14,16,20,25,26,27,30,32,45,46,47]	Los Angeles Abrasion Loss (%)	15–30	28–45	23–35	↑ 5–50% weak mortar contributes to breakage	↑ slight bitumen cushions aggregate during impact
[20,34,40,42]	Aggregate Crushing Value (%)	9.3–22.0	15.5–30.0	15.0–25.0	↑ moderate mortar fracture under load	↑ slight bitumen absorbs compressive energy
[20,37,45,48,49]	Aggregate Impact Value (%)	10–20	16–25	16–22	↑ slight–moderate	↑ slight
[12,15,24]	Aggregate Abrasion Value (%)	19–24	28–45	23–36	↑ higher poor surface hardness due to mortar	↑ moderate bitumen softens under friction at elevated temp.
[22,24,26,29,45]	Particle Shape	Angular to sub-angular (crushed)	Angular; rough, irregular old mortar exposed on surface	Angular to sub-angular smooth bitumen-coated surface	More irregular surface texture; higher angularity	Smooth, hydrophobic surface; lower surface roughness
[7,9,34,50,51,52,53]	Asphalt/Bitumen Content (%)	None	None (trace cement dust only);	CRAP: 2.17–3.04% FRAP: 3.04–5.60%	N/A	FRAP has 2–3× more binder than CRAP; binder aging reduces ductility and bonding
[2,12,22,24]	Adhered (Residual) Mortar Content (%)	None	20–45% by mass (depends on crushing degree and parent w/c)	None	Primary cause of elevated porosity, absorption, and ITZ weakness in RCA	N/A
[6,8,10,12,27,37,50,54,55,56,57]	Surface Texture/Wettability	Hydrophilic; rough surface promotes bond	Hydrophilic; rough, porous; strong mechanical interlocking	Hydrophobic bitumen repels water and cement paste; contact angle > 90°	Better mechanical interlocking but weak ITZ from mortar microcracks	Poor chemical bonding; bitumen layer inhibits C-S-H formation at interface
[6,10,11,50,55,57]	ITZ Configuration	Single ITZ (paste–aggregate)	Double ITZ: ① new paste → old mortar ② old mortar → original aggregate	Single but pervasive weak zone: cement paste → hydrophobic bitumen coating	Double ITZ creates two planes of weakness; ITZ width increases with RCA content	Single hydrophobic interface limits C-S-H bond; failure mode depends on mortar grade and binder aging
[6,8,12,52]	ITZ Thickness & Quality	Thin, dense ~5–20 µm	Thicker, porous ~20–50 µm; microcracks from crushing	Thick, discontinuous bitumen layer up to several µm	ITZ thickens proportionally with RCA replacement; w1, w2 parameters increase	Bitumen layer impedes hydration product formation; thicker void-rich zone than NA
[7,11,43,44,50,52,56]	Failure Mechanism at Aggregate Interface	Cohesive fracture through paste or aggregate	Fracture through old mortar or at ITZ1/ITZ2 planes; microcracking dominant	Cohesive (within cement mortar) for M10–M20; adhesive (at mortar–asphalt interface) for M30–M40; aging asphalt → cohesion failure	Mechanical interlocking governs; surface treatment can heal ITZ	Failure mode is a function of mineralogy, binder aging, and mortar grade
[7,19,22,47,54,58,59]	Microcracking	Minimal	Pre-existing microcracks from crushing and original hydration	None intrinsic; but bitumen may develop thermal cracking at low temperature	↑ higher crack density; governs stiffness loss and permeability	Low crack density; bitumen seals any microfractures
[22,24,60]	Drying/Autogenous Shrinkage	Low (reference)	Moderate–high; higher if RCA used dry (not pre-wetted); pre-wetted RCA can reduce autogenous shrinkage by 45–78%	Low–moderate; bitumen restrains volumetric change; RAP shows less shrinkage than RCA in concrete	↑ higher if not pre-saturated; can become ↓ if pre-wetted (internal curing effect)	↓ lower; bitumen binder restricts free shrinkage
[18,48,54,61]	Freeze–Thaw Durability	Good	Moderate; depends on parent concrete quality and ITZ density	Good; bitumen acts as flexible sealant protecting aggregate	↓ reduced with high RCA; carbonation or surface treatment improves	RAP–concrete shows good resistance; UHPGC with RFA demonstrates good freeze–thaw resistance
[20,34,40,42]	Sulfate and Chloride Resistance	Good	Moderate; higher permeability from mortar porosity	Poor in sulfate-rich environments (dramatic losses in RAP–RCCP in SO_4_^2−^/Cl^−^)	↓ reduced; microcracks allow ion ingress	↓ significantly reduced for RAP–RCCP; high risk in aggressive chemical environments

Note: ↑ indicates an increase, while ↓ indicates a decrease.

**Table 2:** Summary of compressive strength outcomes for RCA- and RAP-based concrete systems across reviewed studies.

Ref.	Study/Material	Replacement (%)	CS (Mpa)	Change vs. Control
[47]	RCA → CRCA (UHPC)	Varied (A&A model)	122.66	+9.1% vs. natural aggregate
[7]	RFA carbonated (UHPC)	0–30%	~same as control at 20%	−12 to +5% (1d, 7d, 28d)
[62]	MCRCF (UHPC)	~50%	118 MPa (28d A2 group)	+3.9%
[63]	RCP as cement sub. (UHPC)	10–70%	129.9–164.5 Mpa	−17.6% to +4.4%
[64]	RCA 50% (UHPC)	50%	146.9 MPa (base)	~−3% at 28d
[50]	RFA (UHPC)	0–100%	Decreases with RFA%	−13.3% at 100% RFA
[65]	Fine RA (UHPC)	100%	159.34 → 184.91 Mpa	−3.1% (no GO); +16% with GO
[58]	RFA particle sizes (UHPC)	20%	160.6 MPa at UCR3-20	+7.1% at 28d
[13]	RAP concrete	10–30%	~25 MPa max at 28d	Decreases with RAP%
[26]	RAP & RCA columns	20–100%	RAP: ~25 MPa; RCA: ~28.1 Mpa	Decreases with replacement
[18]	RAP in RCCP	50–100%	≥27.6 MPa (all except 100% total RAP)	~5% reduction
[19]	RAP geopolymer	25–100%	56.7 MPa (control); 32.5 MPa at 25% RAP	−42.8% at 25%
[41]	RAP in SCC	0–100%	Decreases proportionally	−19.6 to −45.9%
[23]	RAP + steel fibers	20–60%	Increases then decreases	Optimal ~20–40% RAP
[29]	RAP + bagasse ash	50–100%	27.2–35.3 MPa at 28d	−13 to −43% at 90d
[24]	RAP structural concrete	Varied	Decreased with RAP%	Consistent reductions
[14]	RAC + RAP RC beams	20–40% RAC; 10–40% RAP	21–23 MPa	Decreases with recycled content

**Table 3:** Summary of the effect of RCA and RAP on tensile strength and applied improvement methods.

Ref.	Replacement Level	Tensile Strength Effect	Improvement Method
[13]	10%, 20%, 30% RAP; 20%, 40%, 60%, 100% RCA; RAP–RCA blends	Splitting tensile strength decreased progressively with increasing RAP and RCA replacement at all temperatures (20–500 °C). At 20 °C, RAP reduced tensile strength by 8%, 18%, and 28% for 10%, 20%, and 30% replacement. RCA reduced tensile strength by 3–14% at 20 °C and by 16–44% at 500 °C.	None evaluated
[65]	100% fine RA replacing natural river sand	100% fine RA replacement reduced direct tensile strength of UHPC by 8.43% (from 7.95 to 7.28 MPa) relative to natural sand control.	Graphene oxide (GO) addition at 0.02–0.08 wt%
[23]	20%, 40%, 60% RAP	Split tensile strength decreased progressively as RAP percentage increased at both 7 and 28 days; maximum split strength was 3.3 MPa at 0% RAP (28-day, no fibers).	Steel fibers (hooked end) at varying fractions
[29]	50% and 100% CRAP or FRAP	Splitting tensile strength reduced linearly with RAP content (R^2^ > 0.9). At 28 days, 100% CRAP and 100% FRAP reduced tensile strength by 26% and 44%, respectively, compared to control. 50% CRAP and 50% FRAP produced equivalent tensile strength.	Sugarcane bagasse ash (BGA) replacing 10% or 15% OPC
[38]	30%, 50%, 100% RCA	Tensile strength (fct,sp) was the concrete property most significantly affected by RCA replacement. Slabs with ρ = 0.7% showed approximately 8% reduction; slabs with ρ = 1.2% showed approximately 30% reduction. Reduction linked to inherent quality variability of RCA, not directly to replacement ratio.	None evaluated

**Table 4:** Summary of the effect of RCA and RAP on flexural strength and improvement methods.

Ref.	Replacement Level	Flexural Strength Effect	Improvement Method
[67]	0%, 50%, 100% RA	Flexural strength (fp) decreased as RA substitution rate increased: peak load fp decreased most significantly at 100% RA. Increasing NS content further reduced flexural strength (e.g., from 7.02 to 4.67 MPa at 50% RA, 1% SF, as NS rose from 0 to 5%).	Steel fibers (SFs) 1–2%; nano-silica (NS) 1–5%
[64]	100% RCA replacing NCA	UHPC-RCA exhibited lower flexural strength than UHPC-NCA. The RCA’s old mortar created uneven stress distribution in the tension zone, promoting stress concentration and early crack propagation.	Carbon nanofibers (CNFs) 0.25–1%; steel fibers (SFs) 0.5–2% (single and hybrid)
[11]	25%, 50%, 100% RA (untreated URA)	Untreated RA reduced flexural strength by 11.9%, 14.7%, and 20% at 25%, 50%, and 100% replacement, respectively, relative to natural aggregate control (21.8 MPa).	Physical/chemical surface treatment of RA (T1RA, T2RA, T3RA); straight and hooked steel fibers (SSF, HSF)
[65]	100% fine RA replacing natural river sand	100% fine RA reduced flexural strength from 17.14 MPa (natural sand) to 15.94 MPa (−7.0%), attributed to numerous RA interfacial transition zones and weakened properties of adhered old mortar.	Graphene oxide (GO) 0.02–0.08 wt%
[66]	40%, 60%, 80% RAP; 80% RCA–20% RAP, 60% RCA–40% RAP, 40% RCA–60% RAP, 20% RCA–80% RAP	Unheated RAP beams: flexural load reduced by 6.7–13.4% vs. control (139.1 kN). RCA–RAP beams at normal temperature: 20% RAP and 60% RAP mixes showed ~20% reduction; 40% RAP and 80% RAP mixes up to 42% reduction. At 600 °C, RCA–RAP beams lost 55–65% of flexural capacity.	None evaluated
[19]	25%, 50%, 100% RAP	25% RAP: flexural strength reduced by 26.2% (6.17 MPa → ~4.55 MPa). 50% RAP: 40.2% reduction. 100% RAP: 42.2% reduction. Rate of flexural strength loss was less severe than compressive strength loss (72.3% at 100% RAP).	None evaluated
[19]	25–100% RAP (with 5–25% crumb rubber)	Increasing RAP content significantly reduced flexural strength of RCCP. Regression: ft = 3.39 + 11.98C − 3.20RAP − 35.13R^2^ (R^2^ = 0.90). Weak adhesion between RAP particles and cement mortar was the principal cause; unlike compressive loading, tension offered no compensating alignment benefit.	Crumb rubber at 5% (marginal strength benefit)
[40]	100% RCA	100% RCA concrete (RAC): modulus of rupture fct,fl = 4.2 MPa vs. 4.5 MPa for NC (mean values). RAC beams exhibited crack widths up to 5% wider and deflections up to 30% larger than NC beams due to high RCA porosity weakening concrete-bar bond.	Steel fibers 1% by volume
[24]	Up to 40% RAP	Flexural strength decreases as RAP content increases. A 40% RAP mix with w/c = 0.53 showed 37% reduction in flexural strength vs. 52% reduction in compressive strength at 28 days. 20% RAP: flexural strength 14% lower than control, but flexural toughness 48% higher.	None evaluated
[44]	20% and 50% RCA	Without fibers: 20% RCA concrete fL ≈ 65% and 50% RCA concrete fL ≈ 60% of reference fL. The presence of recycled aggregate reduced the limit of proportionality fL compared to natural aggregate concrete.	Steel fibers 20, 35, 50 kg/m^3^

**Table 5:** Summary of effect of RCA and RAP on shear strength of RC members.

Ref.	Replacement Level	Direct Effect on Shear Strength	Improvement Method
[14]	RAC: 20–100%; RAP: 10–30%; RAP–RAC blends	RAC beams: increasing RCA content reduced load-carrying capacity; RAC-only beams lost ~15% capacity at 400 °C vs. control. RAP mixes: increasing RAP content increased load capacity due to rough RAP surface enhancing aggregate–paste bond. RAP + RAC under 400 °C: significant combined capacity loss.	None
[27]	RAP: 20–100%; RAP–RCA: 80% RAP–20% RCA → 20% RAP–80% RCA; RCA: 20–80%	RAP group: shear capacity increased as RAP decreased—154.2 kN (100%), 231.5 kN (60%), 252.1 kN (20%); 55–63% gain vs. RAP100. RCA group: capacity fell from 140.2 kN (20%) to 128 kN (80%) due to weak ITZ, old mortar, microcracks, and high absorption. RAP–RCA group: RCA content was dominant—higher RCA decreased shear.	None
[49]	100% RCA	100% RCA reduced shear cracking strength by 14.5% and ultimate shear capacity by 15.6% vs. NA; pre-cracking stiffness fell 13.68%. RCA accelerated crack formation and widened cracks (49.7% wider at ultimate load).	3D, 4D, 5D hooked SF at Vf = 0.25–0.75%
[24]	CRA: 30%, 50%, 70%, 100%	CRAC beams showed enhanced shear strength vs. untreated RAC beams. Shear strength decreased significantly only near 50% CRA replacement, then change slowed above 70%. Concrete shear contribution (Vc) increased with CRA rate.	CO_2_ carbonation surface treatment of RCA
[51]	5–100% CRA	Contradictory results due to adhered mortar. 30% CRA had a reasonably less detrimental impact on shear strength. fcRA/fcNA ratio stayed within ±10% up to 30% CRA. High-strength concrete beams more sensitive to CRA replacement.	Various (review scope)
[56]	100% RCA	100% RCA reduced shear strength by 12% on average. RAC beams showed less ductile failure than NA counterparts due to weaker aggregate–paste bond.	GFRP vs. steel stirrups
[74]	RAC (recycled aggregate concrete)	Unstrengthened NAC and RAC control beams had comparable shear force values. CFRP strengthening produced a higher % increase in shear for RAC than NAC beams.	CFRP laminates (various configurations)
[30]	100% RCA; 60% RCA + 40% NCA	60% RCA + 40% NCA uniaxial shear capacity was ~8% higher than both 100% NCA and 100% RCA. 60% RCA blend outperformed 100% RCA under all load inclinations. Biaxial capacity had a quadratic relationship with load inclination angle.	Partial replacement (60% RCA blend)
[32]	RCA up to 100% (database)	Increasing RCA content drastically decreased shear strength across the 401-beam database. Fracturing through RCA preferred over NA due to two ITZs, reducing aggregate interlock via smoother fracture surfaces.	None (predictive study)
[69]	RCA 0–100% (database)	30% RCA reduced SBC by 11–19%. 100% RCA: 11% reduction. 100% fine + coarse RCA: 30% reduction. Code DBJ61/T88-2014 underestimated capacity more severely at higher RCA ratios.	None (predictive study)
[72]	RCA: 0%, 50%, 100%; RFA: 100%	At 1.0% SF and 100% RFA, shear capacity fell by 10.43% (50% RCA) and 15.52% (100% RCA) vs. 0% RCA baseline. Both RFA and RCA increases reduced shear capacity.	Steel fibers (Vf = 1.0%)
[61]	RCA: 0%, 30%, 50%; CR: 0%, 5%, 10%	Above 30% RCA, adding PP fiber decreased ultimate shear strength. CR reduced ultimate shear capacity. 50% RCA with or without additives adversely affected shear.	Polypropylene (PP) fiber 1% by volume
[43]	RCA: 0%, 30%, 70%, 100%; T: 20–600 °C	Elevated temperature reduced shear capacity and stiffness. Within ≤600 °C, increasing RCA (via equivalent total water method) initially raised capacity; above 600 °C, ITZ was destroyed, eliminating this benefit. Increasing stirrup spacing from 100 → 200 mm reduced shear by ~24.5%, 22.9%, and 20.7% at 20 °C, 400 °C, 600 °C, respectively.	None
[70]	Rubber recycled aggregate concrete (lower tension zone grade: 22 or 30 MPa vs. 40 MPa control)	FE simulations showed the top higher-grade concrete layer plays no role in shear resistance. Shear resistance of two-layer beam must be calculated using the lower concrete grade. Lower shear resistance of rubber recycled aggregate concrete had minimal implication on beam shear, as shear is mainly resisted by shear links.	Two-layer beam configuration (structural strategy)
[46]	100% RAC	Unstrengthened NAC and 100% RAC beams showed virtually identical shear capacity—maximum 5% difference. RAC beams are viable shear alternatives to NAC.	CFRP U-wraps
[52]	Multi-RAC: 1, 2, 3 recycling cycles (n)	Shear strength degraded with increasing recycling cycles (n) when normal stress was present (σ/fc ≠ 0). At σ/fc = 0, shear strength was limitedly varied with n. Failure mode shifted from typical shear to combined shear-axial collapse as σ/fc rose to 0.8. Reduced contact friction and aggregate interlock were primary degradation mechanisms.	None
[3]	Coarse RA: 30–100%; fine RA: various	Shear strength decreased by 11–19% at 30% coarse + fine RA replacement. At 100% coarse RA replacement, shear decreased by 11%. Using both fine and coarse RA at 100%: 30% reduction—most severe outcome. No significant difference in deflection or ultimate shear between RA and NA beams at low replacement ratios.	None
[2]	RCA and RAP: 50% and 100%	50% RCA reduced shear by 9% (G1.6) and 4.66% (G2.7); 50% RAP by 11.7% (G1.6) and 7.52% (G2.7) vs. NCA. RAP caused larger shear reductions than RCA at same replacement. At 100% replacement with modified mix design (higher cement), shear capacity of RCA and RAP beams equaled NCA. RCA beams more brittle; RAP beams more ductile.	Modified mix design (increased cement content for 100% replacement)
[34]	RCA: 0%, 25%, 50%, 75%, 100%	100% RCA replacement level reduced shear strength (push-off) by 17.3%. Shear strength decreased progressively with rising RCA content due to reduced fracture toughness and aggregate interlock of RCA. FEA and DIC agreed within 5%.	None
[68]	RCA: 0–100%; RCG: 0–20%	Shear capacity of RC beams decreased as RCA content increased, except for 50% RCA + 20% RCG mix, which showed a 5% improvement. Beam with 100% RCA + 20% RCG had shear capacity nearly equal to the control mix.	Recycled crushed glass (RCG) as fine aggregate partial substitute
[57]	RCA: 0%, 50%, 100%	RCA replacement ratio had minimal effect on shear performance of SRRAC short beams. Shear-span ratio was the dominant factor: decreasing λ from 1.52 to 1.14, and 0.76 increased shear bearing capacity by 32.1% and 78.1%, respectively.	Section steel (I14/I16)—structural reinforcement strategy
[73]	RPET–SCBA combined (no direct variation of RCA level)	SCBA–RPET beams showed a shear capacity 17.38% higher than conventional beams, despite 11% lower flexural capacity. Crack patterns during shear and flexural tests were similar and comparable between SCBA–RPET and conventional beams.	5% SCBA (cement substitution) + 10% RPET (sand substitution)
[30]	100% RCA; 60% RCA + 40% NCA	60% RCA + 40 %NCA uniaxial shear capacity was ~8% higher than both 100% NCA and 100% RCA. 60% RCA blend outperformed 100% RCA under all load inclinations. Biaxial shear capacity had a quadratic relationship with load inclination angle.	Partial replacement blend (60% RCA)
[71]	RAC web core (100% in tension zone); NAC flange/outer shell	Shear resistance of two-layer semi-precast beams must be calculated using the lower concrete grade. Shear links dominate shear resistance; lower shear resistance of RAC in the tension zone had minimal implication on total shear capacity. Precast RAC blocks were highly effective at controlling flexural-shear crack development.	Precast RAC block construction approach

**Table 6:** Summary of failure modes observed in RCA and RAP concrete members.

Ref.	Replacement Level (%)	Failure Mode (Unstrengthened/Baseline)	Improvement Strategy
[15]	20–100% RAC; 10–30% RAP	All beams failed in brittle manner via dominant diagonal shear crack—identical failure pattern for RAC, RAP, and NA beams regardless of replacement ratio. Failure initiated at mid-span flexural crack then propagated to diagonal shear crack.	None
[64]	50% RCA	50% RCA in UHPC produced slightly lower compressive strength (−2.8%) and marginally inferior post-peak behavior vs. NCA–UHPC. RCA had a positive effect on reducing compressive strength loss at elevated temperatures.	Carbon nanofibers (CNF) 0.25–1 wt%; steel fibers (SF) 0.5–2 wt%; hybrid CNF + SF
[13]	N/A (failure mechanism study)	Failure mode in RAP concrete governed by aggregate mineralogy and asphalt aging. Granite: adhesive failure at aggregate–asphalt interface. Limestone/sandstone at ≤M20: cohesive failure in asphalt layer; at >M20: adhesive at mortar–asphalt interface. Aged asphalt: predominantly cohesive failure. Physical bonding mainly via Van der Waals interactions.	Higher asphalt grade/aging (material characteristic)
[9]	30%, 50%, 70%, 100%	All beams failed by shear compression failure; main diagonal crack connected loading point to support. Wider cracks near loading point narrowing toward support. Failure pattern consistent across all CRA replacement ratios.	CO_2_ carbonation treatment of RCA
[56]	100% RCA	Two failure modes observed: (1) shear failure—vertical cracks at soffit propagating diagonally to loading point; (2) combined shear/flexural failure with major diagonal crack. NA beams more ductile than RCA beams due to stronger aggregate–rebar bond.	GFRP stirrups vs. steel stirrups
[74]	100% RAC	Unstrengthened NAC and RAC beams: critical diagonal shear crack at 45°. Failure modes of NAC and RAC control beams were comparable.	CFRP laminates (U-wraps, side-bonded, continuous wrapping)
[31]	100% RCA; 60% RCA blend	All specimens failed by diagonal tension failure in shear span. Failure mode consistent across NCA, RCA, and 60% RCA + 40% NCA groups under all load inclinations. Flexural-diagonal cracks formed at mid-shear-span then propagated to critical diagonal crack.	Partial RCA blend (60% RCA + 40% NCA)
[77]	20%, 60%, 100%	Unstrengthened beams with 20% RCA: shear-flexural failure. Shear crack width increased with RCA content—5.6 mm (20%), 6.5 mm (60%), 6.9 mm (100%) vs. 4.9 mm (NA control). RAC beams experienced more brittle shear behavior.	CFRP sheets (2 or 4 continuous layers)
[61]	0%, 30%, 50% RCA	Beams without PP fiber (with CR) failed in brittle mode along wider shear crack at ultimate load. Beams with 50% RCA (with or without additives) showed brittle failure. Higher crumb rubber content increased number of cracks and promoted brittle failure.	PP fiber (1% by volume)
[44]	0%, 30%, 70%, 100%	RRAC beam failure modes similar to RNAC beams at all tested temperatures—no explosive spalling during heating. Elevated temperature degraded shear capacity and stiffness but did not alter the fundamental failure type.	None (elevated temp. study)
[79]	60% treated RCA	Beams without fibers (RASF0) and with 0.5% fibers (RASF0.5): shear failure. First crack load for RASF0: 49.05 kN; ultimate load: 166.7 kN.	Steel fibers at 0.5%, 1.0%, 1.5% Vf; pozzolan slurry surface treatment of RCA
[47]	100% RAC	Unstrengthened NAC and RAC beams: diagonal shear crack failure at 45° from horizontal (from support to loading point). NAC and RAC had virtually identical failure modes and shear capacity (≤5% difference).	CFRP U-wraps
[52]	Multi-cycle recycling (*n* = 1,2,3)	At low σ/fc: typical shear failure. As σ/fc increased to 0.8: failure shifted to complex combined shear-axial collapse. σ/fc = 0.8 dominated by axial collapse regardless of concrete type or n. Increasing n (recycling cycles) flattened post-peak softening curve—failure became more gradual.	None
[35]	0%, 25%, 50%, 75%, 100%	All specimens: Mode II (shear) fracture failure. During initial loading, crack width barely changed; at higher loads, shear crack opened rapidly with slip after inflection point. For RCA > 30%, shear transfer strength reduced by ≥15%. 100% RCA reduced shear strength by 17.3%.	None; new fracture mechanics equation proposed
[26]	100% RCA	Failure mode not affected by aggregate type (GA vs. RCA)—all beams failed in tension-controlled flexure (steel yielding). RCA beams had a higher number of closely spaced cracks and 14.3% lower cracking load vs. GA beams.	20% fly ash (FA) replacement of OPC
[78]	30% RCA	30% RCA concrete had 24% higher compressive strength than conventional aggregate. Unretrofitted RCA beams reached 57% higher load capacity than conventional RC beams. Load-deflection behavior of unretrofitted RCA beams outperformed conventional beams.	GFRP laminates (Scheme 1: full zone; Scheme 2: X-shaped strips) + spiral transverse reinforcement

**Table 7:** Summary of studies investigating the effect of RCA and RAP on workability of fresh concrete.

Ref.	Study Context	Replacement Level	Workability Effect
[48]	UHPC with RCA (particle size 2.36–4.75 mm) and CO_2_ carbonation treatment; slump flow and setting time tested	RCA partial replacement of NA in UHPC	Direct addition of RCA reduced flowability and setting time of UHPC, attributed to higher water absorption and surface roughness of RCA particles.
[12]	UHPC with untreated RA (URA) and three surface modification treatments (acid washing T1, cement slurry coating T2, chemical strengthening T3); slump and flowability tested	25%, 50%, 100% RA	Untreated RA (URA): irregular cracks and voids on aggregate surface absorbed free water, and frictional resistance between irregularly shaped particles further hindered flowability, reducing workability of concrete.
[42]	SCC with RAP at 0–100% replacing NCA; slump flow and T500 tests; DIC crack analysis	RAP 0%, 30%, 60%, 90%, 100%	RAP had a clear detrimental impact on SCC workability. Slump flow ranged between 680 mm and 510 mm across all RAP contents; T500 time ranged from 3.2 s to 7.6 s with increasing RAP content, indicating longer flow time (reduced flowability) at higher RAP levels.
[24]	RAP at 0–100% + steel fibers (SFs) at 0–1.5% in conventional RC concrete; BS EN slump test	RAP 0%, 20%, 40%, 60%, 80%, 100%	Workability of fresh concrete decreased as RAP percentage increased, caused by asphalt mortar coating on RAP aggregates, dirt particles, and irregular shape of RAP aggregates. At 20% RAP, workability further reduced as steel fiber content increased, reaching minimum at 1.5% SF.
[30]	RAP concrete with coarse RAP (CRAP) at 50–100% and fine RAP (FRAP) at 50–100%; BGA at 10–15% replacing cement; ASTM C143 slump tests	CRAP: 50%, 100%; FRAP: 50%, 100%	RAP reduced workability significantly. NA control: 15.5 mm slump. 100% CRAP: 11 mm (−29% vs. NA). 100% FRAP: 0 mm (−100% workability). FRAP had a far greater negative effect than CRAP. The high viscosity of asphalt film coating on RAP aggregates and hygroscopic BGA particles further increased water demand.
[68]	SCC with RCA (0–100%) and recycled crushed glass (RCG) as fine aggregate substitute (0–20%); slump flow, T500, L-box, and V-funnel tests	RCA: 0–100%; RCG: 10–20%	Control SCC mix: slump flow 695 mm (T500 = 2.4 s). 50% RCA + 10% RCG (M-G10-R50): 640 mm; 50% RCA + 20% RCG (M-G20-R50): 655 mm; 100% RCA + 10% RCG (M-G10-R100): 600 mm; 100% RCA + 20% RCG (M-G20-R100): 625 mm. RCA consistently decreased slump flow due to old mortar absorbing free water. V-funnel time slightly increased with RCA; L-box blocking ratio reduction was insignificant.

**Table 8:** Summary of investigating the effect of recycled aggregates on porosity of concrete.

Ref.	Study Context	Replacement (%)	Effect on Porosity
[63]	UHPC with recycled concrete powder (RCP) and recycled paste powder (RPP) replacing cement or silica fume (SF); water-permeable porosity (vacuum saturation) and MIP paste porosity measured	RCP (recycled concrete powder); RPP (recycled paste powder) 10%, 30%, 50%, 70% replacing cement or SF	Cement replacement: RCP at 10–70% raised water-permeable porosity by 27.1%, 68.8%, 92.0%, and 148.2%, respectively; RPP caused larger increases of 37.6%, 100.1%, 181.0%, and 286.4%. SF replacement: RCP at 10–70% increased water-permeable porosity by 12.0%, 31.7%, 41.9%, and 73.4%; RPP by 27.8%, 50.9%, 69.3%, and 99.4%. MIP paste porosity: Control-CEM + SF = 1.88%, SF-70RCP = 9.52%, SF-70RPP = 13.40%; average pore diameter: 11.26 nm, 13.90 nm, 22.57 nm, respectively. RCP produced finer pore structure than RPP at equal replacement.
[50]	UHPC with recycled fine aggregate (RFA) at 0–100% replacing NFA; ITZ parameters (w_1_, w_2_, w_3_) and pore microstructure quantified; standard curing (SC) and autoclaved curing (AC) compared	Recycled fine aggregate (RFA) 0%, 25%, 50%, 75%, 100%	RFA has inherently higher porosity than NFA due to the old cement matrix. Introduction of RFA directly weakened overall UHPC density. More RFA → more total ITZ length and area (w_1_ and w_2_ increased); comprehensive microstructural parameter w_3_ decreased, confirming progressive weakening of the average microhardness of all material phases. The mechanical properties degraded almost linearly with increasing w_1_ and w_2_.
[23]	Characterization of 5 Italian RAP types (AN, AR, BO, MA, PI) as concrete aggregate: open porosity by MIP (10 mm coarse particles), contact angle, freeze–thaw resistance	100% RAP aggregate characterization	MIP open porosity: AN = 5.4%, AR = 3.3%, BO = 6.5%, MA = 5.3%, PI = 6.1%—all exceeding the natural aggregate (NA) average of 2.7%. Pore size distributions showed noteworthy microporosity contribution. Hydrophobic bituminous coating (contact angle 130–135° vs. ~40° for NA granite) impeded water penetration, requiring 48 h for SSD. Most porous RAP (BO, PI): lowest frost resistance (F2/FEC4); AN, AR, MA: F1/FEC2. Post-freeze–thaw WA increased with progressive bituminous layer removal.
[19]	RCCP with coarse RAP (RC), fine RAP (RF), and combined RAP at 50% and 100% replacement; total permeable voids (TPV) at 28 and 91 days; durability in sulfate and chloride environments	Coarse RAP, fine RAP, combined RAP 50% and 100% of each fraction	All RAP fractions (coarse, fine, combined) reduced total permeable voids of RCCP relative to NA control at both 28 and 91 days—the same trend as water absorption. At 28 days: 100% coarse RAP −29%, 100% fine RAP −6%, 100% combined RAP −38% vs. control. Mechanism: asphalt film on coarse RAP melted during boiling test, infiltrating capillary voids within the concrete matrix and reducing porosity. Existing methods for porosity and water absorption determination are not valid for RAP-inclusive specimens.

**Table 9:** Summary of studies investigating the effect of recycled aggregates on freeze–thaw resistance.

Ref.	Study Context	Aggregate	Direct Effect on Freeze–Thaw Resistance
[60]	UHPGC with 100% RFA (metallurgical slag, 905 kg/m^3^); steel fiber at 0–3 vol%; precursor component adjusted (GGBS/FA/RHA ratio); 300 rapid F–T cycles (−20 °C to +20 °C, GB/T 50082-2009)	RFA (metallurgical slag, 100% replacing natural sand)	All UHPGC specimens (100% RFA) showed good freeze–thaw resistance after 300 cycles. Compressive strength loss and mass loss both remained low. Strength loss decreased as steel fiber content increased: UHPGC-S3 (3 vol% SF) had 10.69% lower strength loss than UHPGC-S0 (0 SF) after 300 cycles. Optimal precursor (RHA-enriched, UHPGC-R2) achieved lowest compressive strength loss of 5.82% among all mixes after 300 cycles. Mass loss was relatively marginal, suggesting inner microstructure damage is more serious than surface exfoliation.
[65]	UHPC with 100% fine RA replacing NFA + GO (0–0.08 wt%); 300 F–T cycles; remaining mass and relative dynamic elastic modulus measured (GB/T 50082-2009) [81]	Fine recycled aggregate (100% replacing NFA)	100% fine RA (U0) slightly reduced F–T resistance vs. natural sand control (UR): remaining mass of U0 after 300 cycles = 99.22% vs. UR = 99.37%; relative dynamic elastic modulus of U0 = 95.93% vs. UR = 96.54%. Both metrics ranked U0 below UR, confirming fine RA marginally deteriorates F–T resistance due to higher porosity and weaker old mortar at ITZs.
[23]	Five Italian RAP types (AN, AR, BO, MA, PI) subjected to 10 F–T cycles in water (EN 1367-1) and NaCl solution (EN 1367-6); mass loss and classification per EN 12620	RAP aggregate (5 Italian sources)	F–T mass loss varied with RAP source porosity. AN, AR, MA: F = 0.89–0.96%, classified F1 (≤1%) in water and FEC2 in NaCl—good frost resistance. BO: F = 1.63%, FEC = 2.25%—class F2/FEC4 (poor). PI: F = 1.96%, FEC = 3.27%—class F2/FEC4. BO and PI were the most porous (6.5% and 6.1% MIP open porosity), confirming porosity as the primary F–T durability risk factor. Post-F–T water absorption increased progressively as the bituminous layer was partially removed.

**Table 10:** Summary of CO_2_ reduction and environmental impact of RCA/RAP in concrete.

Ref.	Study Context	Material	CO_2_/Environmental Impact Finding
[8]	UHPC with 20% carbonated RFA; LCA-based GWP evaluation compared to UHPC without RFA	Carbonated RFA (20% of sand mass)	Use of 20% carbonated RFA in UHPC enabled a 5% decrease in Global Warming Potential (GWP) relative to UHPC without RFA, evaluated by life cycle assessment. The carbonation pre-treatment absorbs CO_2_ into the RFA matrix, and the replacement of cement-intensive mixes with RFA further reduces embodied carbon. The same 20% carbonated RFA also reduced UHPC cost by more than 15%.
[83]	UHP–ECC with RCP replacing cement (C), GGBS (G), and silica sand (S) at 0–100%; carbon footprint (kg CO_2_-eq/m^3^) and unit cost quantified	Recycled concrete powder (RCP) in UHP–ECC	Carbon footprint of RCP = 0.001 kg CO_2_/kg vs. 0.83 kg CO_2_/kg for cement. C75 (75% cement replaced by RCP) reduced carbon footprint by 61.7% vs. reference. C50G100S100 (50% cement + 100% GGBS + 100% silica sand replaced by RCP) reduced carbon footprint by 48.3%. RCP cost = $0.03/kg vs. cement $0.048/kg.
[5]	EIO-LCA sustainability assessment of three RAP–PCC pavement types vs. plain PCC; GHG, energy, water, air pollutants quantified	RAP as coarse aggregate in PCC pavement (single-lift and two-lift)	Single-lift RAP–PCC: petroleum-based fuel use −13.86%; CO_2_ fossil −0.72%; NOx −3.13%; PM10 −11.52%; water withdrawals −6.70%; human health non-cancer −37.87% vs. plain PCC. Two-lift RAP–PCC (RAP in bottom lift): CO_2_ fossil −3.97%; greater social and environmental benefits. Aggregate cost comprises 20–30% of concrete pavement material cost; use of RAP reduces virgin aggregate extraction.
[18]	Economic assessment of RAP aggregate production chain in Italy; cost compared to natural aggregate (NA) and recycled concrete aggregate (RCA)	RAP as structural concrete aggregate (Italy)	Current RAP aggregate production cost is +155.39% higher than NA, driven by acquisition (milling + recycling fees) and transport distance. Transformation and assessment phases of RAP are 41.91% and 31.80% cheaper than NA equivalents. Simultaneously acting on all three critical operations (acquisition, end-of-waste process, and transport) could reduce RAP cost by 39.64% vs. NA, and by 45.13–67.30% vs. RCA.

**Table 11:** Summary of LCA findings for RCA and RAP in concrete and pavement systems.

Ref.	Study Context	Material	LCA Findings (Own Data)
[6]	Systematic review: RAP in rigid concrete pavements; LCA context; sustainability gaps identified; optimal RAP proportions recommended	RAP as natural aggregate substitute in PCC	The study identifies that RAP use reduces waste product volume and disposal burden on landfills and favors cost-effectiveness and environmental preservation over virgin materials. Research gaps are highlighted on LCA of RAP pavement systems—specifically the lack of comprehensive LCA data for RAP in rigid concrete and the need to integrate circular economy principles. The optimum RAP proportion is recommended based on sustainability balance with strength retention.
[5]	Economic Input-Output LCA (EIO-LCA) of single-lift RAP–PCC, two-lift RAP–PCC, and plain PCC pavement; full life cycle inventory; TRACI impact assessment	RAP in Portland cement concrete (PCC) pavement	EIO-LCA showed single-lift RAP–PCC yielded highest economic benefit (−10.90% total economic impact vs. plain PCC) and two-lift RAP–PCC (RAP in bottom lift) yielded highest social and environmental benefit. Energy use: −1.25% (single-lift), −4.13% (two-lift). Water withdrawals: −6.70% and −9.20%, respectively. Smog: −3.05% and −6.21%. Total greenhouse gases: +3.00% (single-lift), −1.30% (two-lift)—with the two-lift design achieving net GHG reduction.
[84]	LCA of multi-generation recycled aggregate concrete (MGRAC) beams up to 3 recycling generations; GWP per m^3^ quantified; virgin aggregate savings calculated	RCA through 1, 2, and 3 recycling generations (MGRAC)	GWP (kg CO_2_-eq/m^3^): VA = 358.65, RA-1 = 353.82, RA-2 = 353.75, RA-3 = 353.67—all three MGRAC generations showed minor GWP reduction vs. virgin aggregate concrete. The reduction in GWP is small because the variation is driven by processing emissions differences only. However, each recycling generation saves 1060 kg/m^3^ of virgin aggregate, and waste concrete is diverted from landfill for at least 3 generations—representing a significant ongoing material savings benefit not fully captured by GWP alone.

**Table 12:** Summary of cost-effectiveness and economic savings from RAP use in concrete.

Ref.	Study Context	Material	Cost-Effectiveness/Economic Savings Finding
[24]	RAP at 0–100% in RC concrete + steel fibers; cost savings calculated per m^3^ vs. NA control (Uganda scenario)	RAP (0–100% replacing NA)	Maximum cost savings of 6.13% was achieved at 60% RAP replacement vs. the natural aggregate control mix. At 20% RAP replacement, cost reduction was 1.64%. Higher RAP content reduced material cost because RAP is available as a waste product at near-zero material cost (transportation cost only). RAP provides a long-term solution for reducing construction material costs and end-of-life disposal burden in landfill.
[5]	EIO-LCA cost analysis of RAP–PCC pavement; aggregate cost as % of material cost; full economic activity quantified	RAP as coarse aggregate in PCC pavement	Aggregate cost comprises 20–30% of concrete pavement material cost. Single-lift RAP–PCC reduced total economic impact by 10.90% vs. plain PCC. RAP haul cost ($155,171) was lower than virgin aggregate haul ($379,333 for plain PCC, $318,472 for two-lift RAP–PCC) due to shorter transport distance and local availability. Landfill diversion further reduced waste management costs ($17,734 saved for single-lift).
[30]	RAP (CRAP + FRAP) concrete with 10–15% BGA as cement substitute; economic analysis of 1 m^3^ concrete cost vs. conventional concrete (Indian market)	RAP (coarse + fine) + BGA (10–15% cement replacement)	Incorporating RAP aggregates blended with 10% BGA reduced the total cost of 1 m^3^ concrete by more than 40% compared to conventional concrete. RAP and BGA are both locally available waste materials at negligible raw material cost (transport cost only assumed). Higher RAP proportions progressively reduced concrete cost. The 10% BGA recommendation also improved mechanical and durability properties.
[18]	Four-step cost evaluation of RAP aggregate production chain (Italy); comparison vs. NA and RCA per m^3^ of structural concrete	RAP as structural concrete aggregate (Italy)	RAP aggregate unit cost is currently +155.39% higher than NA due to acquisition (asphalt milling + recycling fees) and transport costs. However, transformation (−41.91%) and assessment (−31.80%) phases of RAP are cheaper than NA equivalents. Acting simultaneously on all three critical operations (acquisition, end-of-waste process, and transport) reduces RAP cost by 39.64% vs. NA—making RAP competitive—and 45.13–67.30% cheaper than RCA.

## Data Availability

No new data were created or analyzed in this study. Data sharing is not applicable to this article.

## Supplementary Materials

The following supporting information can be downloaded at https://www.mdpi.com/article/10.3390/ma19173601/s1, Table S1: PRISMA 2020 Checklist.

## Abbreviations

RCA	Recycled Concrete Aggregate
RAP	Reclaimed Asphalt Pavement
SF	Steel Fiber
CNF	Carbon Nano-Fibers
ITZ	Interfacial Transition Zone
UHPC	Ultra-High-Performance Concrete
NA	Natural Aggregate
UCRCA	Uncarbonated Recycled Concrete Aggregate
CRCA	Carbonated Recycled Concrete Aggregate
ACRCA	Accelerated Carbonation Recycled Concrete Aggregate
RAC	Recycled Aggregate Concrete
NAC	Natural Aggregate Concrete
LCA	Life Cycle Assessment
GHG	Greenhouse Gas
RCCP	Roller-Compacted Concrete Pavement
GFRP	Glass Fiber-Reinforced Polymer
CFRP	Carbon Fiber-Reinforced Polymer
SEM	Scanning Electron Microscopy
DIC	Digital Image Correlation

## Appendix A

**Table A1:** Characteristics and key findings of studies investigating recycled coarse and fine aggregate (RCFA) and reclaimed asphalt pavement (RAP) in structural concrete applications.

Ref. No.	Author (s) (Year)	Variables	Tested Parameter	Investigation	Output
[1]	Abdel-Jaber et al. (2026)	Recycled coarse and fine aggregate (RCFA) replacement ratio; exposure temperature.	RCFA replacement ratio: 0%, 25%, 50%, 75%, 100%. Temperature: 23 °C, 400 °C, 600 °C.	Experimental and finite element investigation of the shear performance of reinforced concrete beams incorporating simultaneous recycled coarse and fine aggregates after fire exposure.	Ultimate shear capacity decreased by 6–10% with increasing RCFA content at room temperature and by up to ~22% after exposure to 600 °C. Moderate RCFA replacement (25–50%) preserved acceptable residual shear performance while improving ductility and energy absorption.
[2]	Soltanabadi and Behfarnia (2022)	RCA/RAP content (50–100%), deep beam shear strength, cracking patterns.	Shear capacity, energy absorption, cracking pattern.	Shear strength of deep beams containing natural coarse aggregates (NCAs), recycled concrete aggregates (RCAs), and recycled asphalt pavements (RAPs).	100% RCA/RAP mix achieved equivalent shear strength to natural aggregate beams after mix design modifications.
[3]	Al Mahmoud et al. (2020)	Coarse and fine aggregate replacement ratio, crack propagation, shear load capacity.	Shear capacity, crack distribution, modulus of elasticity.	Shear behavior of reinforced concrete beams made from recycled coarse and fine aggregates.	Shear capacity reduced by 10–20% with higher RCA/RFA content. Crack widths were wider in recycled concrete beams.
[4]	Shi et al. (2018)	RAP content in PCC (0–100%), cost assessment, environmental footprint (CO_2_ emissions), social benefits (employment impact).	Economic savings, CO_2_ reduction, environmental benefits, material life cycle.	Performing a sustainability assessment of Portland cement concrete (PCC) pavement containing RAP aggregates using life cycle inventory analysis and economic input–output analysis.	RAP–PCC offers highest economic savings and CO_2_ reduction, making it a viable alternative to traditional pavement materials.
[5]	Rout et al. (2023)	Effects of RAP content on pavement mechanical strength, durability, microstructural stability, and its role in sustainable construction.	Strength, durability, microstructure properties.	Comprehensive and insightful evaluation of the utilization of RAP aggregates based on characterization, strength, durability, and microstructure properties along with life cycle analysis (LCA).	RAP shows potential for sustainable pavement applications, though strength reductions must be mitigated with additives or design adjustments.
[6]	Lu et al. (2024)	Bond-slip performance, concrete–rebar interface behavior, cyclic loading response.	Pull-out strength, bond stress, interfacial transition zone strength.	Dynamic performance of components constructed from recycled concrete incorporating aggregates modified by accelerated carbonation.	Accelerated carbonation improved bond-slip performance by 7.6% compared to untreated recycled concrete.
[7]	Huang et al. (2023)	Carbonated vs. uncarbonated RFA, hydration, mechanical properties, sustainability impact.	Pore refinement, shrinkage, cost-effectiveness, hydration kinetics.	Influence of carbonated recycled fine aggregate (RFA) on mechanical and microstructural performance of ultra-high-performance concrete (UHPC). This includes the changes in strength, hydration kinetics, RFA-to-paste interfacial microstructure, and pore structure with the use of carbonated RFA.	Carbonated RFA reduced autogenous shrinkage by 45% and enabled 5% reduction in global warming potential.
[8]	Zhou et al. (2024)	Carbonation modification levels, RCA replacement ratio (30–100%), shear strength, shear deflection.	Shear strength, shear deflection, diagonal crack width.	Examining the effects of carbonation modification on the shear performance of recycled aggregate concrete beams.	Carbonation modification improved RCA shear performance, but replacement ratios above 70% led to strength reduction.
[9]	Zhao et al. (2023)	Polypropylene fiber content, crack formation, peak load, drying shrinkage.	Peak load, crack width, drying shrinkage strain, mechanical properties.	Assessment of flexural response of RC beams and unrestrained shrinkage of fiber-reinforced high-volume fly ash-based no-aggregate concrete and self-compacting concrete.	Polypropylene fibers delayed first crack formation and improved peak load in no-aggregate concrete beams.
[10]	Luo et al. (2023)	Water-glass concentration, ITZ structure, durability, compressive strength.	ITZ bond strength, water absorption, flexural strength, fracture toughness.	The use of water glass to immerse silicomanganese slag (SS) was investigated as a method for optimizing the interfacial transition zone (ITZ) in ultra-high-performance concrete (UHPC).	Water glass treatment optimized ITZ, increasing mechanical strength and durability.
[11]	Sun et al. (2024)	RA treatment method, workability, strength, porosity, ITZ quality.	Compressive strength, flowability, flexural strength, nano-silica interaction.	the influence of modified RA on the workability, mechanical properties, and microstructure of ultra-high-performance concrete (UHPC).	Chemical treatment improved ITZ and enhanced UHPC performance with treated recycled aggregates.
[12]	Bhardwaj and Singh (2024)	Mineralogical characteristics of aggregates, asphalt binder aging, interfacial bond strength in cement–mortar systems.	Surface energy, bond strength, asphalt adhesion.	Analyzes the failure mechanisms of RAP-based concrete through surface-free energy concepts and interfacial bond strength testing.	Failure mechanisms in RAP–concrete varied with asphalt binder aging and aggregate mineralogy, influencing overall durability.
[13]	Abedalqader et al. (2021)	RAP/RCA replacement ratios, compressive strength, modulus of elasticity, stress–strain behavior.	Compressive strength, stress–strain curve, tensile strength, modulus of elasticity.	Influence of temperature on mechanical properties of recycled asphalt pavement aggregate and recycled coarse aggregate concrete.	RAP and RCA decreased mechanical properties at elevated temperatures, but performance remained acceptable for high-temp applications.
[14]	Alrajfi et al. (2021)	Shear capacity, temperature exposure, RCA/RAP content, crack propagation.	Shear strength, failure mode, deflection, residual strength at elevated temperatures.	The structural performance of RC beams made with natural aggregate (NA), recycled aggregate concrete (RAC), and reclaimed asphalt pavement concrete (RAP) under normal and elevated temperature.	Shear capacity of RCA beams was slightly lower than NAC beams, but RAP inclusion further reduced strength at elevated temperatures.
[15]	Arshad and Ahmed (2017)	RAP content (50% and 75%), RCA presence, resilient modulus variation, constrained modulus effects, cyclic strain response.	Resilient modulus, constrained modulus, accumulated strain, stress levels.	Investigating the feasibility of characterization of blended materials containing RAP with fresh granular materials and RCA to evaluate whether they are suitable for granular base/subbase layers of flexible pavements.	Higher RAP content increased resilient modulus but also led to greater residual strain accumulation.
[16]	Naser et al. (2022)	RAP/RCA content, asphalt mix stability, flow, volumetric properties, and optimum asphalt content.	Marshall stability, flow, volumetric properties.	The performance of hot mix asphalts (HMA) with RAP and RCA, in terms of their Marshall stability, flow, and volumetric properties, to verify their applicability as a replacement for the natural aggregate in the flexible pavement surface layers of HMA mixtures.	75% RAP in asphalt improved stability; however, RCA content increased optimal asphalt content, affecting volumetric performance.
[17]	Peduzzi et al. (2023)	Bond strength, RCA percentage, galvanized iron fiber volume.	Bond strength, machine learning accuracy, mix optimization.	Improve the mechanical properties and bond behavior of natural aggregate concrete (NAC) and recycled aggregate concrete (RAC) by incorporating locally available galvanized iron fiber (GIF).	Machine learning models predicted bond strength with >95% accuracy. 0.5% GIF increased bond strength by 46.5%.
[18]	Debbarma et al. (2019)	RAP fraction type (coarse, fine, mixed), mechanical strength, water absorption, sulfate and chloride resistance.	Strength, porosity, sulfate/chloride resistance.	The optimum fraction of RAP (coarse, fine, and total) along with its optimum proportion (50% and 100%) for roller compacted concrete pavement (RCCP) mixes, based upon various fresh, mechanical, and durability properties.	50% RAP maintained RCCP strength while reducing costs by 46%, but durability concerns in sulfate-rich environments remain.
[19]	Albidah (2023)	RAP percentage, compressive/flexural strength, strain at peak strength, temperature resistance at elevated exposure levels.	Compressive/flexural strength, strain at peak strength, temperature resistance.	The potential of producing metakaolin-based geopolymer concrete incorporating reclaimed asphalt pavement (RAP) aggregate of five concrete mixes with 0%, 25%, 50%, and 100% coarse RAP aggregate replacing the natural aggregate.	25% RAP reduced strength by 42.8% but enhanced strain at peak strength, indicating improved ductility.
[20]	Karthikeyan et al. (2023)	Concrete durability, mechanical properties, sustainability aspects, feasibility of RAP substitution, environmental impacts.	Strength and durability metrics, cost-effectiveness of RAP in pavement.	Analyzing the potential, benefits, and limitations of using RAP in concrete pavement construction and assessing strength and durability parameters in comparison to conventional concrete.	Identified need for improving RAP concrete strength and durability. Further research required for optimizing mix designs.
[21]	Masi et al. (2022)	RAP particle size distribution, porosity, water absorption, durability under freezing–thawing cycles.	Water absorption, porosity, microstructure, freeze–thaw durability.	Physical properties, microstructure by microscopy, dimensional stability, and durability of reclaimed asphalt pavement (RAP) sourced from 5 different Italian collections.	RAP aggregates exhibited higher porosity but can be modified to meet concrete performance requirements.
[22]	Andrew et al. (2022)	RAP replacement ratio (0–60%), steel fiber content (0.5–2%), curing time (7–28 days), concrete workability, compressive strength.	Compressive strength, split tensile strength, workability, cost savings.	The mechanical performance of reclaimed asphalt pavement (RAP) along with steel fibers in concrete and the variation of mechanical behavior concerning different curing times with the optimal RAP aggregate substitute ratio.	Maximum RAP replacement ratio of 60% achieved 6.13% cost savings. Higher RAP content reduced workability due to asphalt coating effects.
[23]	Jaawani et al. (2021)	RAP content in structural concrete, compressive strength, flexural strength, Young’s modulus.	Compressive strength, durability, flexural strength.	Evaluating the limitations on the use of recycled asphalt pavement in structural concrete.	RAP concrete met structural standards but exhibited lower strength and durability limitations.
[24]	Abushanab and Alnahhal (2022)	Mixing water type, RCA ratio, fly ash content, flexural strength.	Flexural capacity, crack pattern, load-deflection behavior.	Flexural behavior of reinforced concrete beams prepared with treated wastewater, recycled concrete aggregates, and fly ash.	TWW and RCA decreased flexural capacity by 13.7% and 15.9%, respectively, while fly ash increased ductility and load capacity.
[25]	Shatarat et al. (2019)	Axial capacity, crack propagation, RCA/RAP ratio, theoretical vs. experimental results.	Axial load capacity, crack width, strain distribution, concrete mix properties.	The axial compressive behavior of fifteen reinforced concrete columns that were constructed from four types of aggregates: natural aggregate (NA), recycled asphalt pavement (RAP), recycled coarse aggregate (RCA), and RAP–RCA.	Columns with higher RAP and RCA content showed reduced axial capacity, but experimental values exceeded theoretical predictions.
[26]	Arabiyat et al. (2021)	Shear force, deflection, cracking pattern, RAP/RCA content.	Shear capacity, crack width, shear span-to-depth ratio.	Shear behavior of thirteen reinforced concrete (RC) beams made of recycled asphalt pavement (RAP) and recycled coarse aggregate (RCA).	RAP replacement reduced shear capacity, but RCA showed improved performance at lower replacement levels.
[27]	Rahardjo et al. (2013)	Particle size distribution, permeability, water-entry value, shear strength, porosity, compaction level.	Permeability, water retention, shear strength in saturated and unsaturated states.	Studying the unsaturated properties of RAP and RCA, including permeability, water characteristic curves, and shear strength, for potential geotechnical applications.	RAP has lower permeability and higher suction retention than natural aggregates, making it suitable for landfill covers and drainage layers.
[28]	Singh et al. (2018)	RAP content (50–100%), bagasse ash (10–15%), compressive strength, cost reduction, workability, durability improvement.	Workability, compressive and tensile strength, cement reduction efficiency.	Evaluating the feasibility of utilizing RAP aggregates mixed with sugarcane bagasse ash (BGA) for producing concrete, assessing mechanical and durability properties.	10% bagasse ash in RAP concrete improved strength and reduced cost by 40% compared to conventional concrete.
[29]	Aldmour et al. (2023)	Load inclination angles, RCA percentage (60% vs. 100%), shear force-deflection behavior.	Biaxial shear capacity, load inclination impact, failure mode.	Examining the biaxial shear behavior of recycled concrete aggregate reinforced concrete beams.	60% RCA and 40% NCA mix had higher shear capacity than 100% RCA under all load inclinations.
[30]	Hung et al. (2024)	pH range (3–11), DOC levels (low to high), liquid-to-solid (L/S) ratio (2–10), metal concentrations (Pb, Zn, Cu, Ni), compaction level.	Metal concentration in leachates, effect of pH and DOC on leaching behavior.	Metals leaching characteristics from RCA and RAP due to the variations in key influential factors of pH, dissolved organic carbon (DOC), compaction, and liquid to solid ratio (L/S).	Acidic conditions (pH < 5) increased metal leachability. Higher compaction reduced metal mobility in leachates.
[31]	Jayasinghe et al. (2023)	Shear strength, RCA replacement ratio, reinforcement type, beam geometry.	Shear capacity, machine learning prediction accuracy, SHAP analysis.	Using machine learning to predict the shear strength of recycled aggregate concrete beams with and without shear reinforcement.	XGBoost achieved 95% accuracy for slender beams and identified RCA replacement ratio as a minor factor in shear strength.
[32]	Elsayed et al. (2023)	RCA ratio (0–100%), aluminum fiber content (0–3%), WGP replacement (20%).	Load capacity, toughness, stiffness, ductility.	Structural performance of recycled coarse aggregate concrete beams containing waste glass powder and waste aluminum fibers.	Adding waste aluminum fibers improved ductility and load capacity. The optimal fiber content was 1%.
[33]	Imjai et al. (2023)	RCA replacement ratio, shear stress distribution, fracture mode.	Shear strength, push-off failure mode, FEM validation.	The shear behavior of recycled aggregate concrete (RAC) Z push-off specimens with different replacement levels of recycled concrete aggregate.	New equation predicted shear strength with 5% accuracy compared to experimental data.
[34]	Wang et al. (2023)	ECC layer height, shear-span ratio, interfacial bond strength, flexural performance.	Load-bearing capacity, failure mode, ECC contribution to flexural strength.	Flexural performance of 3D-printed composite beams with engineered cementitious composites (ECC) and recycled fine aggregate concrete.	ECC layer improved flexural strength, but bond strength of printed interfaces limited further improvements.
[35]	Sharaky et al. (2023)	Recycled aggregate replacement, steel fiber content, flexural strength, ductility.	Maximum displacement, flexural strength, strengthening efficiency.	the effect of steel fiber-reinforced concrete (SFRC) jacketing on the flexural performance of coarse recycled aggregate-reinforced concrete (CRARC) beams.	Strengthening with SFRC jackets significantly increased flexural capacity and ductility.
[36]	Shahjalal et al. (2023)	PPA percentage, bond strength, shear span-to-depth ratio, flexural toughness.	Ultimate moment capacity, ductility, bond strength, toughness.	Flexural and bond-slip responses of reinforced concrete beams containing recycled coarse aggregate and polypropylene plastic.	5% PPA improved ultimate moment capacity by 28.6% and bond strength by 16.6%, but 10% PPA reduced performance.
[37]	Ferreira et al. (2024)	Slab–column connection type, RCA percentage, punching strength, flexural response.	Punching resistance, modulus of elasticity, tensile strength.	Investigating the punching strength of slab–column connections without shear reinforcement using recycled concrete aggregates.	RCA reduced modulus of elasticity and tensile strength but had little impact on punching resistance.
[38]	Xiamuxi et al. (2024)	RAP percentage, concrete production cost, sustainability factors.	RAP aggregate cost, structural viability, sustainability impact.	economic aspect of RAP aggregate, evaluating the costs associated with its production and comparing them with the ones necessary to produce A and recycled concrete aggregate (RCA).	RAP production cost was 155% higher than natural aggregate but could be reduced by up to 39.64% with optimized processing.
[39]	Imjai et al. (2024)	Beam reinforcement type (steel vs. GFRP), steel fiber content (0–1%), crack width, shear-induced deflection.	Load at first cracking, crack width, deflection, flexural capacity.	Analyzing deflections in high-content recycled aggregate concrete beams reinforced with GFRP bars and steel fibers.	Steel fibers increased first-crack load by 15–17%, and a new Eurocode-based equation improved deflection prediction accuracy.
[40]	Liu et al. (2022)	RAP content, stress–strain behavior, crack propagation, peak stress, peak strain, elastic modulus, energy absorption, and damage variable.	Workability, compressive strength, stress–strain behavior, elastic modulus, crack propagation, toughness index, and constitutive damage model parameters.	The mechanical properties of self-compacting concrete (SCC) with reclaimed asphalt pavement (RAP), SCC samples with RAP content of 0%, 30%, 60%, 90%, and 100% by weight.	Increase in RAP content resulted in reduced compressive strength and workability but improved crack resistance and energy absorption. The damage variable decreased with higher RAP content, improving flexibility.
[41]	Imjai et al. (2023)	Slab RCA content, deflections, crack width, shear-induced deformations.	Deflection, crack width, load-carrying capacity.	Serviceability behavior of FRP-reinforced slatted slabs made of high-content recycled aggregate concrete.	100% RAC slabs exhibited 30% higher deflections than predicted by ACI 440.1R. The FEA model accurately simulated deformations.
[42]	Zheng et al. (2021)	Temperature exposure (200 °C–600 °C), RCA ratio (0–100%), shear strength.	Residual shear capacity, failure mode, stiffness loss.	Assessing the shear behavior of reinforced recycled aggregate concrete beams after exposure to temperatures up to 600 °C.	Shear capacity reduced by up to 40% at 600 °C. Existing design codes underestimated shear strength above 500 °C.
[43]	Costa et al. (2025)	Steel fiber content, recycled aggregate ratio, flexural behavior, ductility.	Flexural strength, crack width, post-cracking behavior.	Studying steel fiber-reinforced recycled aggregate concrete, using SFRRAC as a structural material, and its effect on post-cracking behavior and beam design.	Steel fiber reinforcement improved ductility and reduced crack widths. FEM-based model accurately predicted beam behavior.
[44]	Ashteyat et al. (2024)	Bond strength, load-slip behavior, steel bar diameter, RAP/RCA content.	Bond-slip response, pull-out force, steel bar diameter influence.	The bond strength behavior between steel and concrete made with recycled concrete aggregates (RCAs) and/or recycled asphalt pavement aggregates (RAPs).	Higher RAP content reduced bond stress, with reductions of 6–45% depending on steel bar diameter.
[45]	Abdalla et al. (2022)	Shear capacity, crack pattern, CFRP wrap efficiency, failure mode.	Shear strength, failure load, crack width, deflection.	Comparison of shear behavior of normal and recycled aggregate beams strengthened with CFRP U-wraps.	RAC beams exhibited similar shear capacity to NAC beams. CFRP increased shear strength by up to 60%. ACI code predictions aligned with experimental values.
[46]	Leng et al. (2023)	Carbonation treatment, RCA incorporation, compressive strength, chloride penetration resistance.	UHPC workability, compressive strength, durability, carbonation depth.	Development of ultra-high-performance concrete (UHPC) by carbonated recycled coarse aggregate (CRCA).	Carbonation improved RCA properties, leading to enhanced UHPC strength and durability.
[47]	Fakhri and Amoosoltani (2017)	Crumb rubber content (5–25%), RAP percentage (25–100%), energy absorption, toughness, flexural and compressive strength.	Flexural strength, compressive strength, absorbed energy, toughness index.	Analyzing the effects of incorporating RAP and crumb rubber on the mechanical properties of roller-compacted concrete pavement (RCCP) using regression and ANOVA approaches.	Optimal RAP–rubber mix (10% rubber, 50% RAP) improved toughness and energy absorbency. Higher RAP reduced compressive strength.
[48]	Sheikh et al. (2025)	Steel fiber type (3D, 4D, 5D), volume fraction (0.25–0.75%), shear span-to-depth ratio.	Shear strength, crack propagation, stiffness, ductility.	Studying the effect of 3D, 4D, and 5D steel fibers on the shear behavior of reinforced concrete beams made of recycled coarse aggregate.	100% RCA reduced shear capacity, but steel fibers improved strength by up to 156% with optimal volume fraction of 0.75%.
[49]	Zhang et al. (2018)	Curing condition, recycled fine aggregate content, microstructure parameters.	ITZ thickness, porosity, compressive strength, fracture toughness.	Mechanical behavior of ultra-high-performance concrete (UHPC) using recycled fine aggregate (RFA).	Autoclaved curing improved UHPC strength despite high recycled aggregate content.
[50]	Abdulla (2024)	CRA replacement ratio, beam shear resistance, effect of adhered mortar.	Shear capacity, maximum CRA replacement ratio.	Assessing the role of coarse recycled aggregate in concrete beams and its impact on shear resistance.	30% CRA replacement showed minimal impact on beam shear strength, while adhered mortar reduced performance.
[51]	Lei et al. (2023)	Recycling cycles (1–3), normal stress ratio, mechanical strength, aggregate interlock.	Shear strength, stress–displacement curve, failure mode.	Fracture behaviors of sustainable multi-recycled aggregate concrete under combined compression-shear loading.	Shear strength decreased with recycling cycles but increased with normal stress. Aggregate interlock was a key parameter.
[52]	Li et al. (2024)	Steel tube thickness, RCA replacement ratio, reinforcement ratio.	Flexural capacity, moment-curvature response, reinforcement effect.	The influence mechanism of reinforcement on the flexural behavior of recycled aggregate concrete-filled square steel tube (R-RACFST).	Reinforcement improved the flexural performance of RAC-filled steel tubes. The proposed equation accurately predicted flexural capacity.
[53]	Cheng et al. (2024)	Graphene oxide percentage, permeability, autogenous shrinkage, ITZ properties.	Permeability, pore size distribution, autogenous shrinkage, mechanical strength.	Influence of industrial-grade graphene oxide on macro- and micro-properties of ultra-high-performance concrete incorporating recycled fine aggregate.	Graphene oxide improved ITZ and reduced shrinkage, enhancing durability of UHPC with recycled fine aggregates.
[54]	Guo et al. (2023)	LC3 dosage, RFA content, hydration products, microstructural characteristics, mechanical resistance, and environmental impact.	Compressive, tensile strength, pore structure, nanoindentation, life cycle impact.	The mechanical properties and microstructural characteristics of ultra-high- performance concrete (UHPC) using different dosages of limestone calcined clay cement (LC3) and recycled fine aggregate (RFA).	Using 30% LC3 maintained tensile strength, improved pore structure, and reduced environmental impact via carbon footprint reduction.
[55]	Younis et al. (2022)	RCA replacement ratio (0–100%), shear reinforcement type (GFRP vs. steel), load-carrying capacity.	Shear strength, failure mode, deformation characteristics.	Studying the shear strength of recycled-aggregate concrete beams with glass-FRP stirrups.	Using RCA reduced shear strength by 12%, while GFRP stirrups showed minor differences compared to steel reinforcement.
[56]	Ke et al. (2022)	RCA replacement ratio, shear span-to-depth ratio, section steel type.	Shear strength, stress flow, ultimate load.	Shear bearing capacity of steel-reinforced recycled aggregate concrete short beams based on modified compression field theory.	Shear bearing capacity increased by up to 78.1% as span-to-depth ratio decreased. RCA had little effect on shear strength.
[57]	Chen et al. (2024)	RFA particle size (0–0.6mm, 0.6–1.18mm, 1.18–2.36mm), internal relative humidity, autogenous shrinkage, mechanical properties, and resistance to chloride penetration.	Autogenous shrinkage, internal humidity, compressive strength, chloride penetration resistance.	Feasibility of utilizing recycled fine aggregate (RFA) in ultra-high-performance concrete (UHPC) to alleviate autogenous shrinkage, mitigate environmental effects, and further improve mechanical strengths.	20% RFA (1.18–2.36mm) significantly reduced autogenous shrinkage by 78.5% while increasing compressive strength by 7.1% at 28 days.
[58]	Lin and Wu (2025)	Shear span-to-depth ratio, stirrup type, DCL replacement ratio, crack width.	Shear capacity, diagonal crack width, shear ductility.	Studying the shear behavior of precast recycled lump-aggregate concrete laminated beams using inclined-crossed stirrups.	Inclined-crossed stirrups enhanced shear capacity and reduced crack width compared to traditional stirrups.
[59]	Liang et al. (2023)	Freeze–thaw resistance, compressive strength, microstructure, fiber–matrix bond.	Compressive strength, freeze–thaw cycles, interfacial transition zone structure.	A green ultra-high-performance geopolymer concrete (UHPGC) containing recycled fine aggregate (RFA) was prepared to assess the feasibility of RFA and reveal the reaction mechanism of UHPGC, the reaction process, mechanical properties, freeze–thaw resistance, and microstructure.	Geopolymer concrete with recycled fine aggregate exhibited excellent freeze–thaw resistance and mechanical performance.
[60]	Hossain et al. (2023)	RCA and crumb rubber content, shear strength, crack pattern, toughness.	Shear strength, post-diagonal cracking, deformation behavior.	Examining the shear behavior of polypropylene fiber-reinforced concrete beams containing recycled aggregate and crumb rubber.	Optimal mix of 30% RCA, 5% crumb rubber, and 1% PP fiber improved shear resistance. 50% RCA reduced shear strength.
[61]	Yuan et al. (2024)	Microwave carbonation, RCF incorporation, compressive strength, interface transition zone properties.	Carbonation reaction rate, compressive strength improvement, CO_2_ sequestration efficiency.	Development of ultra-high-performance concrete (UHPC) matrix based on recycled concrete fines subjected to coupling curing of microwave and wet carbonation.	Microwave carbonation enhanced UHPC properties while reducing energy consumption by 26.6%.
[62]	Wu et al. (2024)	Hydration heat, microstructure, mechanical strength, shrinkage, porosity.	Compressive strength, hydration kinetics, porosity, shrinkage resistance.	The multi-performance evaluation of low-carbon UHPC containing recycled powder as a substitution of cement and silica fume.	Recycled powder substitution reduced mechanical strength but improved cement efficiency and environmental sustainability.
[63]	Amin et al. (2022)	Temperature exposure, carbon nanofiber percentage, mechanical properties.	Compressive strength, flexural strength, mass loss, modulus of elasticity.	Influence of recycled aggregates and carbon nanofibers on properties of ultra-high-performance concrete under elevated temperatures.	CNFs enhanced residual strength but increased brittleness at high temperatures.
[64]	Yu and Wu (2020)	GO content, mechanical properties (compressive, tensile, flexural strength), volume stability, durability, and pore structure.	Compressive, tensile, flexural strength, elastic modulus, chloride penetration resistance, freezing–thawing resistance.	The use of graphene oxide (GO) to enhance the properties of ultra-high-performance concrete (UHPC) with fine RA.	GO at 0.06 wt% optimally enhanced UHPC properties, increasing compressive strength by 2.04–16.04%, tensile strength by 7.36–30.50%, and flexural strength by 5.83–23.40%.
[65]	Ashteyat et al. (2024)	RAP/RCA ratio, flexural strength, load-deflection behavior, temperature-induced degradation.	Flexural capacity, load-deflection, temperature effects.	The strength and flexural performance of reinforced concrete (RC) beams incorporating natural aggregate (NA), recycled concrete aggregate (RCA), and reclaimed asphalt pavement concrete (RAP) at normal and elevated temperatures.	RAP + RCA concrete beams showed 55–65% loss in flexural load capacity when exposed to 600 °C.
[66]	Luo et al. (2024)	RA content, nano-silica content, steel fiber amount, flexural response.	Load-deflection response, flexural strength, residual strength.	The feasibility of applying defect-containing recycled aggregates (RAs) in ultra-high-performance concrete (UHPC); studied the effect of nano-silica (NS) and steel fiber (SF) together on its modification.	Steel fibers improved ductility, but nano-silica had a minor negative effect on flexural strength.
[67]	Ahmad et al. (2023)	RCA and RCG percentages, SCC workability, beam stiffness, deflection behavior.	Shear span-to-depth ratio, SCC workability, stiffness.	Effect of recycled crushed glass and recycled coarse aggregate on the properties of self-compacting concrete.	Replacing 20% fine aggregate with RCG improved SCC shear performance. ACI 318-19 provided the best shear capacity estimates.
[68]	Li et al. (2024)	Shear capacity, beam width, hoop reinforcement ratio, compressive strength.	Prediction accuracy, feature importance, sensitivity analysis.	Applying tree-based machine learning models to interpret the shear capacity of reinforced recycled aggregate concrete beams.	Extreme gradient boosting model achieved 96% accuracy, showing hoop reinforcement ratio and beam width as dominant factors.
[69]	Ataria and Wang (2019)	Concrete layer composition, shear resistance, bending capacity.	Shear strength, bending resistance, crack width.	Bending and shear behavior of two layers of different grades of concrete. The top layer (1/3rd) concrete, mainly in compression, is higher grade, and the bottom (2/3rd) layer, in tension, is lower grade using rubber recycled aggregate concrete.	Two-layer beams achieved the same bending resistance as control beams but lower shear resistance due to unzipping effect.
[70]	Fahmy and Idriss (2019)	Basalt fiber length, geopolymer composition, flexural strength.	Flexural strength, tensile strength, crack propagation, deformation.	The effect of hybrid basalt fibers on the mechanical and structural characteristics of geopolymer concrete containing geopolymer-coated recycled concrete aggregates.	Basalt fibers enhanced flexural strength and limited shrinkage cracks in geopolymer-coated RCA concrete.
[71]	Gao et al. (2022)	Steel fiber volume, RCA/RFA replacement ratio, stirrup spacing, mid-span deflection.	Shear strength, mid-span deflection, crack propagation.	Shear behavior analysis and capacity prediction of steel fiber-reinforced concrete beams with recycled fine and coarse aggregates.	Steel fiber content offset shear strength loss due to 100% RCA/RFA replacement. A new shear prediction model was proposed.
[72]	Daniel et al. (2024)	RPET and SCBA content, flexural and shear strength, ductility.	Flexural and shear strength, ductility, energy absorption.	Structural behavior of reinforced concrete beams containing recycled polyethylene terephthalate and sugarcane bagasse ash.	SCBA–RPET beams had 11% lower flexural capacity but 17.38% higher shear capacity than conventional beams.
[73]	Abdalla et al. (2024)	CFRP strengthening configuration, RAC vs. NAC beam performance, failure modes.	Shear force-deflection behavior, CFRP strengthening effect.	Investigating the behavior of normal and recycled aggregate beams strengthened with different types of externally bonded shear reinforcement.	CFRP strengthening was more effective in RAC than NAC beams, improving shear capacity by up to 40%.
[74]	Obaidat et al. (2024)	Precast vs. cast-in-place filler, flexural strength, shear capacity.	Load-deflection response, shear strength, crack width.	Flexural behavior of large-scale semi-precast reinforced concrete T-beams made of natural and recycled aggregate concrete.	Precast RAC blocks improved crack control and deformability in semi-precast T-beams.
[75]	Islam et al. (2025)	Load inclination angle, RCA replacement ratio, shear strength.	Biaxial shear capacity, deformation, failure mode.	Capacity of reinforced concrete beams that include recycled coarse aggregates (RCAs) subjected to biaxial shear loading.	60% RCA and 40% NCA mix had higher biaxial shear capacity than 100% RCA. Load inclination had a quadratic effect on shear strength.
[76]	Mansour et al. (2024)	CFRP strengthening configuration, recycled aggregate ratio, ultimate load, failure pattern.	Load-deflection response, ultimate load, stiffness, FEM validation.	Experimental and numerical evaluation of the shear performance of RAC beams strengthened with CFRP sheets.	Beams with 20% RCA and 4-layer CFRP showed the highest shear capacity (84.8 kN). FEM results confirmed experimental findings.
[77]	Vinod Kumar et al. (2022)	RCA replacement ratio, critical anchorage length, bond strength.	Bond strength, anchorage length, rebar pull-out resistance.	Flexural bond evaluation of deformed steel rebars in recycled aggregate concrete.	RCA weakened bond strength compared to NAC. A new anchorage length modifier proposed for safer designs.
[78]	Mathew et al. (2023)	Steel fiber volume, RCA treatment method, first crack load, ultimate load.	First crack load, failure mode, mid-span deflection.	Investigate the shear behavior of reinforced concrete beams made of treated recycled coarse aggregate (RCA) experimentally after adding three different volume fractions of hook end steel fibers.	1.5% steel fiber shifted failure mode from shear to flexure, enhancing shear resistance in RCA beams.
[79]	Ghalehnovi et al. (2021)	Basalt fiber modification, nano-silica content, flexural resistance, crack propagation.	Peak load, deflection, ductility, strain behavior.	Flexural performance of recycled concrete beams reinforced with modified basalt fiber and nano-silica.	Modified basalt fiber improved bonding properties. Combined with nano-silica, flexural resistance and ductility were significantly enhanced.
[80]	Zhou et al. (2021)	Particle size range, compressive strength, interfacial transition zone properties.	Nanoindentation hardness, strength, pore structure, tensile capacity.	The influence of the particle size range of recycled fine aggregate (RFA) on the microstructural characteristics and mechanical properties of ultra-high-performance concrete (UHPC) was thoroughly investigated by conducting the nano-, micro-, and macro-scale characterization tests.	UHPC incorporating 0–1 mm RFA exhibited superior compressive strength and denser microstructure.
[81]	Liu et al. (2024)	RCP replacement ratio, cement replacement, flexural strength, environmental impact.	Flexural strength, environmental impact, mechanical degradation.	The mechanical properties and environmental benefit of low-carbon UHP–ECC containing high-volume RCP as the replacement of cement, ground granulated blast furnace slag (GGBS), and silica sand.	RCP replacement reduced strength but improved sustainability; optimal balance achieved at 25–50% replacement.
[82]	Visintin et al. (2022)	Basalt fiber volume fraction, prestressing steel strand stress, ultimate bending moment.	Crack width, flexural strength, stress increment in prestressing strands.	Flexural behavior of post-tensioned precast beams manufactured with basalt-fiber-reinforced recycled concrete–conventional concrete.	Basalt fiber reinforcement improved crack resistance and stiffness. Numerical model predicted flexural behavior within 6% accuracy.
